# Characterization and evaluation of cytotoxic and antimicrobial activities of cyclotides from *Viola japonica*

**DOI:** 10.1038/s41598-024-60246-9

**Published:** 2024-04-28

**Authors:** Yuanyuan Lian, Xue Tang, Gehui Hu, Chenfang Miao, Yunfei Cui, Dongting Zhangsun, Yong Wu, Sulan Luo

**Affiliations:** 1 Guangxi Key Laboratory of Special Biomedicine, School of Medicine, Guangxi University, Nanning, China; 2 Key Laboratory of Tropical Biological Resources of Ministry of Education, Hainan University, Haikou, China; 3 Department of Pharmacy, The 900Th Hospital of Joint Logistics Team of the PLA, Fuzhou General Clinical Medical College of Fujian Medical University, Fuzhou, China

**Keywords:** Chemical biology, Plant sciences

## Abstract

Cyclotides are a type of defense peptide most commonly found in the Violaceae family of plants, exhibiting various biological activities. In this study, we focused on the *Viola japonica* as our research subject and conducted transcriptome sequencing and analysis using high-throughput transcriptomics techniques. During this process, we identified 61 cyclotides, among which 25 were previously documented, while the remaining 36 were designated as vija 1 to vija 36. Mass spectrometry detection showed that 21 putative cyclotides were found in the extract of *V. japonica*. Through isolation, purification and tandem mass spectrometry, we characterized and investigated the activities of five cyclotides. Our results demonstrated inhibitory effects of these cyclotides on the growth of *Acinetobacter baumannii* and *Bacillus subtilis*, with minimum inhibitory concentrations (MICs) of 4.2 μM and 2.1 μM, respectively. Furthermore, time killing kinetic assays revealed that cyclotides at concentration of 4 MICs achieved completely bactericidal effects within 2 h. Additionally, fluorescence staining experiments confirmed that cyclotides disrupt microbial membranes. Moreover, cytotoxicity studies showed that cyclotides possess cytotoxic effects, with IC_50_ values ranging from 0.1 to 3.5 μM. In summary, the discovery of new cyclotide sequences enhances our understanding of peptide diversity and the exploration of their activity lays the foundation for a deeper investigation into the mechanisms of action of cyclotides.

## Introduction

Through long-term evolution, plants have evolved a complex defense system, one of which is the production of defense peptides, such as cyclotide. Cyclotides represent a unique class of peptides widely recognized for their distinctive structural attributes and diverse biological activities. Cyclotides are initially identified in *Oldenlandia affinis* of the Rubiaceae family and have since been increasingly found in plants from families such as Violaceae^1–4^, Cucurbitaceae^5^, and Fabaceae^6,7^. A database of Cyclotides, Cybase (http://www.cybase.org.au)^8,9^, contains approximately 1000 cyclotides. These peptides typically range in size from 27 to 38 amino acid residues, presenting a compact and cyclic structure that contributes to their remarkable stability. A key defining feature of cyclotides is the presence of six cysteine residues, forming three pairs of disulfide bonds arranged in a pattern of I–IV, II–V, III–VI, named the cyclic cystine knot (CCK) framework, which is crucial to their structural integrity^10^. Furthermore, the cyclotide’s head-to-tail cyclized structure greatly enhances its resilience^11,12^. Six cysteine residues in cyclotides separate the peptide into six segments, designated as loop 1 to loop 6. Currently, cyclotides can be categorized into three subfamilies, namely Möbius (a Glu in loop 1 and a Pro in loop 5), bracelet (a Glu in loop 1), and squash trypsin inhibitor (squash TI) cyclotide subfamilies. Notably, cyclotides are synthesized within plants through the expression and processing of gene-encoded precursor molecules, showcasing an intriguing biosynthetic pathway that contributes to their unique structural composition and diverse functionalities in various biological environments^1,3,13–15^.

Cyclotides have demonstrated notable thermostability, chemical robustness, and a high level of resistance to enzymatic degradation^16^. Significantly, they exhibit a wide array of biological activities, comprising antimicrobial^17^, antitumor^4,18–20^, insecticidal^21,22^, and hemolytic activities^23^. Furthermore, they show promise in anti-HIV effects^24,25^and enzyme inhibition^5,26,27^. These attributes collectively position cyclotides as intriguing subjects for further exploration and potential applications in pharmaceutical and agricultural domains. The sole cyclotide-containing plant identified in the Fabaceae family is the butterfly pea, whose cyclotide extract has been authorized for agricultural application as an insecticide in Australia^28^. Currently, kalata B1[T20K] is undergoing phase I clinical trials for the treatment of multiple sclerosis, targeting IL-2^10^. Therefore, the discovery of effective plant-derived compounds holds promise for clinical applications. Moreover, due to their unique structure, cyclotides can function as molecular scaffolds to improve drug stability and functionality^29^.

In this study, structural determination and activity analysis were performed on peptides isolated from *Viola japonica*, encompassing assessments of antibacterial activity and cytotoxicity. The discovery of new cyclotides can enhance our understanding of cyclotide evolution and diversity and these new cyclotides may exhibit potential bioactivities or contribute to the determination of cyclotide sequence diversity.

## Results and discussion

### Discovering cyclotides using transcriptome sequencing

Transcriptome sequencing technology, which has become more accessible and affordable due to the rapid development and cost reduction of high-throughput transcriptomics, has been used to explore cyclotides in various plant species. We obtained fresh specimens of *V. japonica* and separated them into three parts: root, stem and leaf, for RNA extraction. We then performed transcriptome sequencing on the high-quality RNA. The raw sequence data reported in this paper have been deposited in the Genome Sequence Archive^30^ in the National Genomics Data Center, China National Center for Bioinformation (GSA: CRA014480) that are publicly accessible at https://ngdc.cncb.ac.cn/gsa.

**Figure 1:**
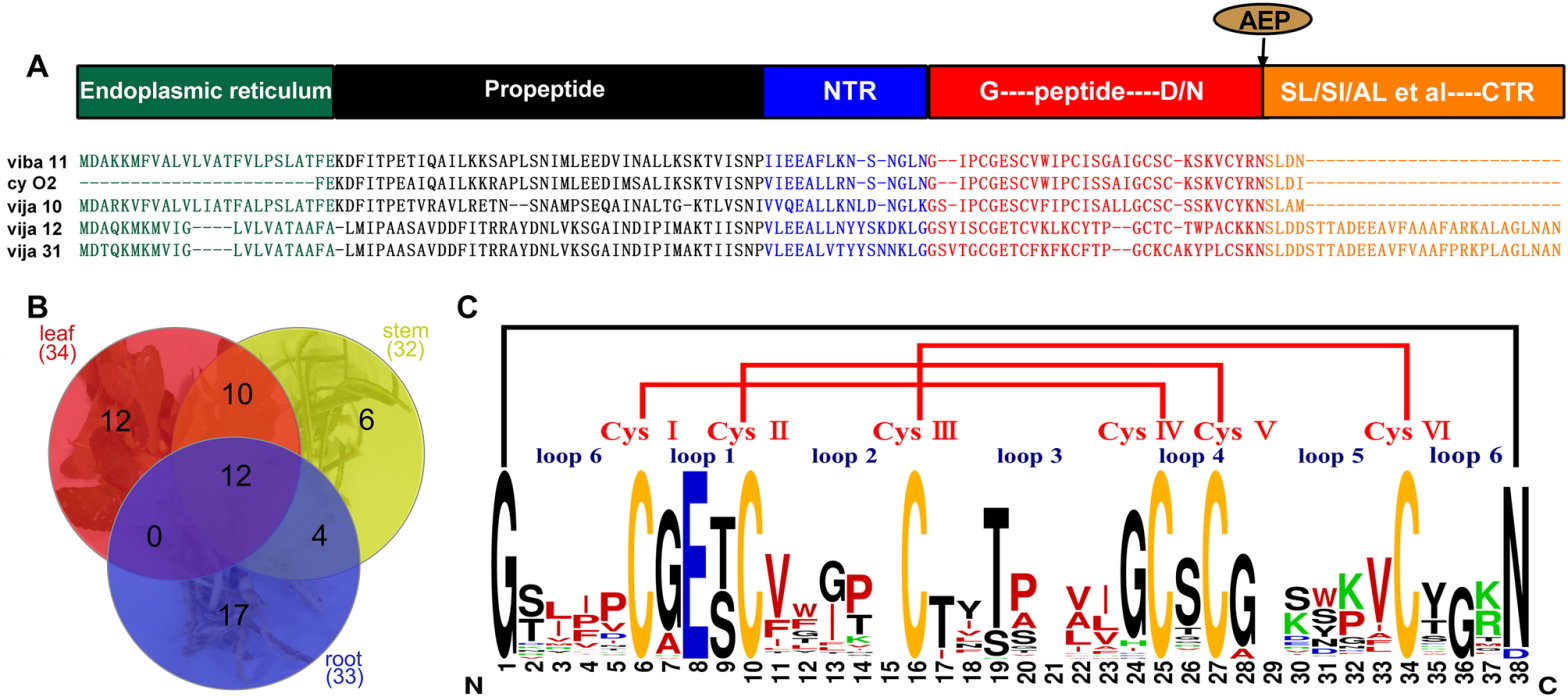
(**A**) The precursor protein of cyclotides typically consists of an ER signal sequence, a propeptide domain, an N-terminal recognition region, a cyclotide sequence, and a C-terminal recognition region. The C-terminal of the cyclotide sequence usually ends in Asp or Asn. These regions serve as recognition sites for asparaginyl endopeptidases, which promote the cyclization of the peptide. The C-terminal recognition region is guided by a small side chain group S/G/A/H and a hydrophobic residue L/I/V^3^. The sequences of 5 cyclotide precursors found in *V. japonica* exhibit this characteristic. (**B**) The distribution of cyclotides in different parts of *V. japonica* (root, stem and leaf) is depicted in a Venn diagram. There are 33 cyclotides in the roots, 32 ones in the stems, and 34 ones in the leaves. (**C**) The amino acid sequence logo representation shows a total of 61 cyclotides found in *V. japonica*. These cyclotides have been aligned, and their sequence is summarized. As depicted in the figure, the cyclotides contain 6 cysteine residues labeled as Cys I–Cys VI. The entire peptide is divided into 6 loops, denoted as loop 1–loop 6. The cysteine residues form 3 pairs of disulfide bonds, indicated by the red lines representing the linkage pattern I–IV, II–V, III–VI. The entire cyclotide is connected head-to-tail, indicated by the black line.

As depicted in Fig. 1A, cyclotide precursors typically comprise five components: the signal sequence of the endoplasmic reticulum (ER), the domain of the cyclotide precursor, and the recognition sequences at the N- and C-termini of the cyclotide sequence, named as NTR and CTR, respectively^3^. More specifically, N-terminal processing by papain-like cysteine protease^13^, then cyclotide is generated by intramolecular ligation mediated by asparaginyl endopeptidases (AEPs). The presence of an Asparagine (Asn; N) or Aspartic acid (Asp; D) at the C-terminus of the cyclotide sequence is essential for AEPs recognition^31–33^. Consequently, cyclotide sequences typically commence with Glycine (Gly; G) at the N-terminus and terminate with D/N, while featuring six cysteines in the middle (Fig. 1A). These sequence characteristics of cyclotide precursors provide a basis for our analysis of transcriptome sequencing.

Through the analysis of transcriptome data, we find cyclotides are present in root, stem and leaf of *V. japonica*, with the highest abundance observed in the leaf. A Venn diagram depicts the distribution of cyclotides in various parts of *V. japonica* (Fig. 1B). In the leaves, 34 cyclotides were identified, while 33 were discovered in the roots, and 32 in the stems. Notably, 12 cyclotides are shared across all plant tissues (root, stem and leaf). Compared with the cyclotide data in the Cybase database, we identified 36 new cyclotide sequences (vija 1 to vija 36) and confirmed 25 previously reported ones (Table S1). According to the cyclotide classification, among the recently discovered cyclotide sequences, 12 belong to the Möbius subfamily (designated as vija 4, vija 9, vija 12, vija 15, vija 18, vija 22, vija 26 to vija 28, and vija 30 to vija 32). The remaining 24 sequences are attributed to the bracelet subfamily, characterized by the absence of Pro in loop 5.The amino acid sequence logo representation^34^ is created (Fig. 1C) based on the alignment of all cyclotide sequences discovered from *V. japonica*. In the chart, blue represents acidic amino acids, green represents basic amino acids, red represents hydrophobic amino acids, yellow represents cysteine, and all other amino acids are depicted in black. Loop 2 and 3 exhibit a higher proportion of hydrophobic amino acids, whereas loops 1 and 4 are relatively conserved. From the figure we can see that three amino acids are very conserved in loop 1, where the second amino acid is all glutamic acid, the first amino acid is glycine or alanine with a short side chain, and the third is threonine and serine which are similar in nature. The most frequently occurring amino acids in loop 2 are valine, glycine and proline. Threonine and glycine at positions 19 and 24 in loop 3 are relatively conserved. The loop 4 only contains one residue, and the most frequent one is serine. The amino acids in loop 5 are relatively variable, and the one that appears more frequently is valine at position 33. Loop 6 is the site recognized by cyclase AEPs, so position 38 is a highly conserved asparagine or aspartic acid, and the first position is a conserved glycine. However, amino acids at other positions in loop 6 are relatively variable.

### Identification of cyclotides using liquid chromatography tandem mass spectrometry

Our strategy for elucidating the cyclotide primary structures of *V. japonica* involved 3 steps, which included (i) preparative liquid phase separation of the crude extract; (ii) reduction and alkylation of each component, followed by MS measurement to analyze the number of disulfide bonds; and (iii) enzymatic digestion and MS/MS sequencing to identify and characterize the primary structure features, combined with transcriptome sequencing results^35^.

**Figure 2:**
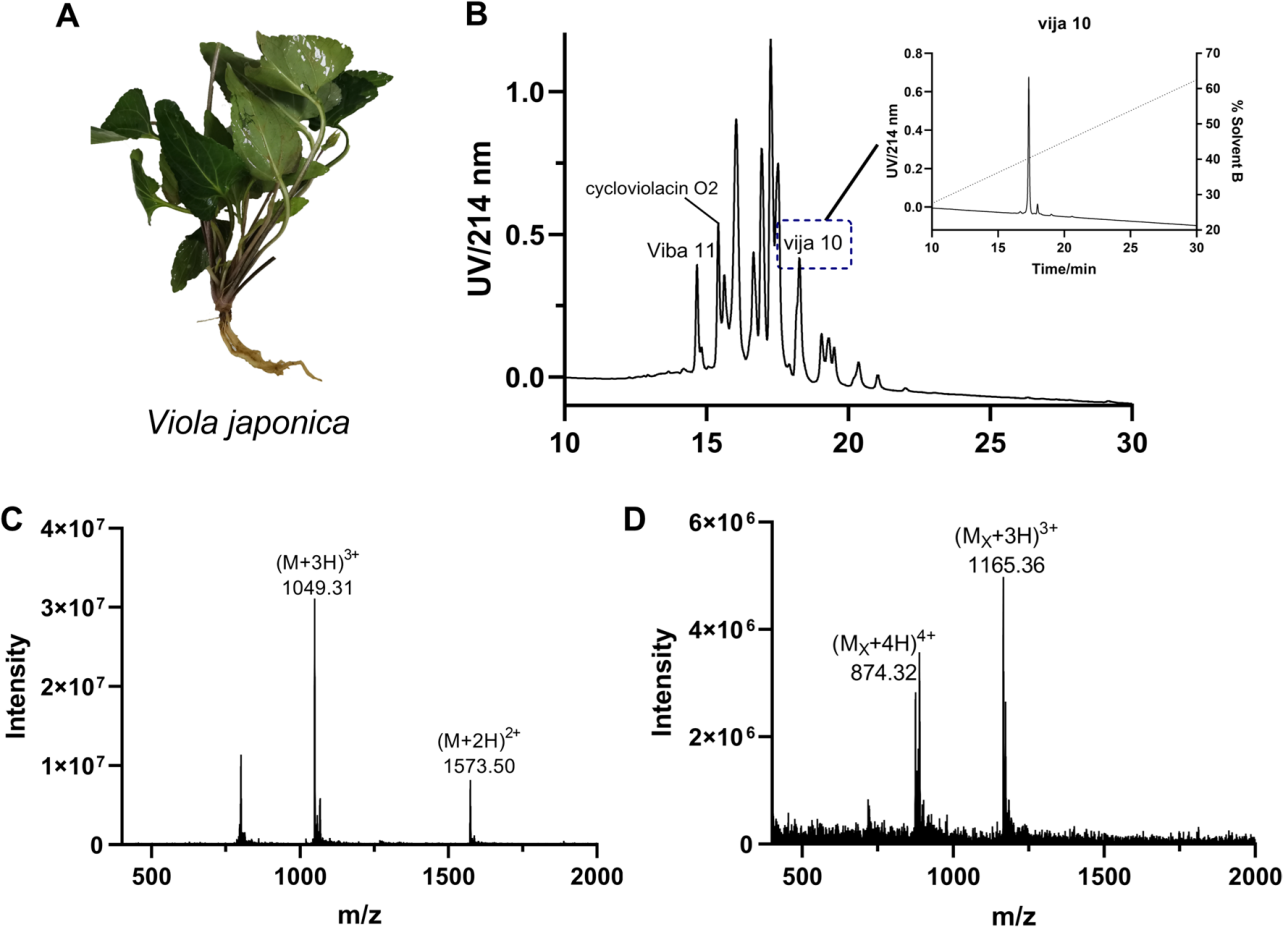
(**A**) Fresh *Viola japonica* (**B**) The chromatogram of the collected solution (1% trifluoroacetic acid in 50% acetonitrile) of *Viola japonica* after purification by SPE extraction. The positions of the peaks corresponding to viba 11, cycloviolacin O2, and vija 10 are indicated. The retention times for these peaks are 14.6 min, 15.4 min, and 18.2 min, respectively. The dotted line depicts the percentage of mobile phase B in the liquid phase conditions. The chromatogram extracted for vija 10 is displayed on the right. (**C**) The mass spectrum of vija 10. (**D**) The mass spectrum of vija 10 after reduction and alkylation.

We collected fresh specimens of *V. japonica* (Fig. 2A), chopped them, and submerged them into 1% formic acid in 50% acetonitrile aqueous solution (1% FA in 50% ACN) overnight for extraction. Firstly, use an SPE-C18 column to fractionate the crude extract using different acetonitrile/water ratios (1%TFA in 30% ACN, 1%TFA in 50% ACN). Then each fraction was injected into the preparative RP-HPLC for further separation. Through mass spectrometry detection of crude extracts, we have identified 21 potentially existing cyclotides which are showed in Table S1. Figure 2B shows the chromatograms of the *V. japonica* extract components (1%TFA in 50% ACN) and the isolation of vija 10 (Fig. 2B). We also isolated viba 11, vija 12, 31 and cycloviolacin O2, with vija 12 and 31 eluting with a 30% acetonitrile–water solution (Fig. S2). We selected each chromatographic peak for mass spectrometry analysis to determine its molecular weight. After that, the components which might contain cyclotides were subjected to dithiothreitol (DTT)-mediated reduction of disulfide bonds, followed by iodoacetamide (IA) treatment to alkylate the exposed cysteine residues and prevent their disulfide bonds reformation. We measured the molecular weight of the reduced and alkylated peptides by mass spectrometry, and calculated the difference between the two weight values to confirm the presence of cyclotides with a mass shift of 348 Da. As an example, Fig. 2C,D illustrate the changes in the molecular weight of vija 10 after the reduction and alkylation reactions (Fig. 2C,D). The molecular weight difference between them is 348 Da, consistent with our prediction.

**Figure 3:**
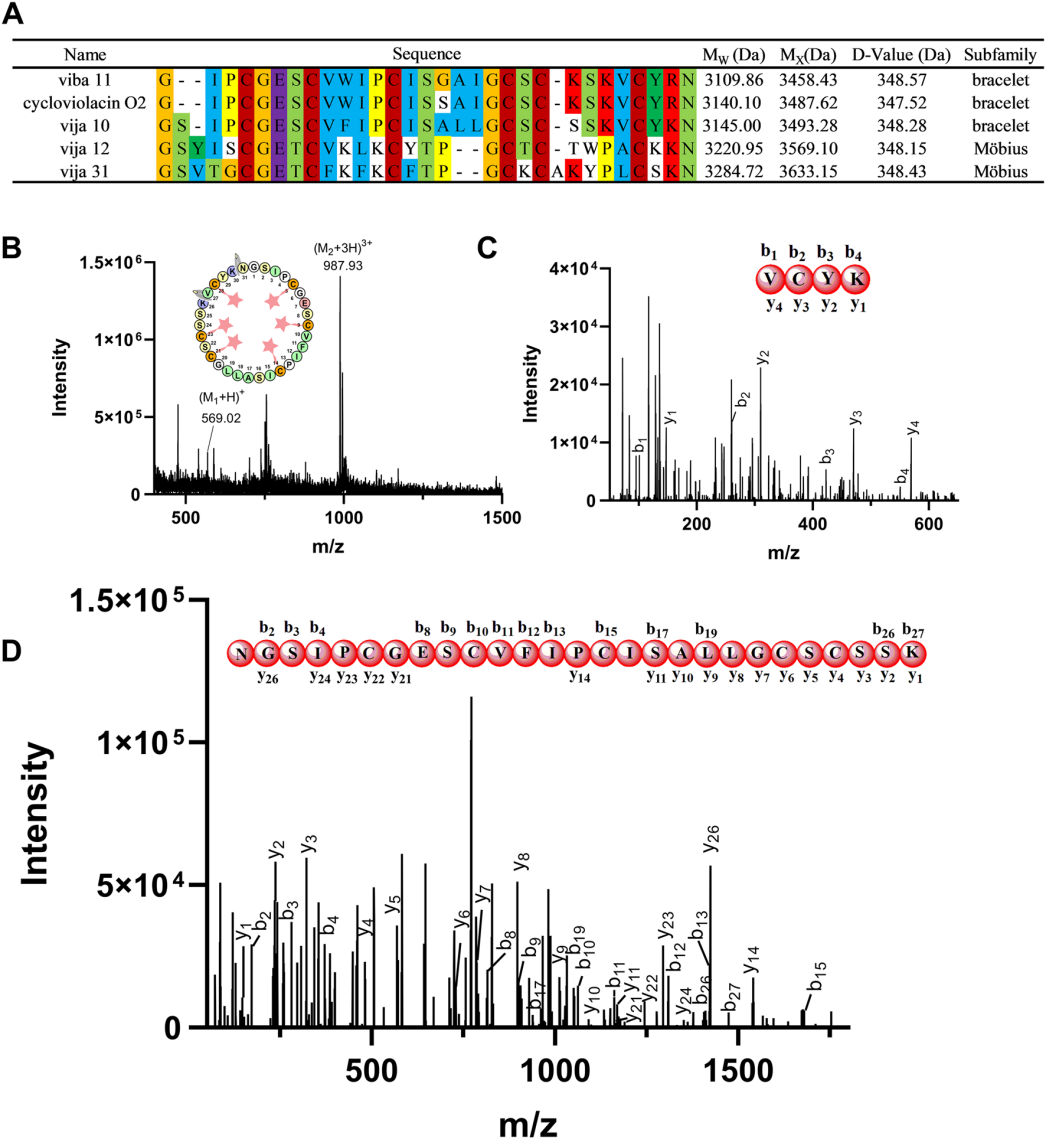
(**A**) Cyclotides extracted from the *Viola japonica*, along with their sequence, and subfamily. M_w_ represents the observed molecular weight (M), M_x_ represents the reduced and alkylated molecular weight (M_x_), and D-value indicates the difference between them. (**B**) Determination of vija 10 sequences by enzymatic digestion tandem mass spectrometry. After reduction and alkylation, the cyclotide is digested by trypsin, with cleavage occurring at the carboxyl groups of lysine and arginine residues. Since the cyclotide contains two lysine residues, it is cleaved into two fragments with precursor masses of 2961.14 Da and 569.02 Da. (**C**) and (**D**) The enzymatically-digested fragments which were analyzed through tandem mass spectrometry. The observed b and y ions are marked in bold on the linearized sequence.

In the same way as described above for the identification of vija 10, we utilized isolation and purification, reductive alkylation and mass spectrometry with respect to identification, etc., we isolated and purified the cyclotides viba 11, cycloviolacin O2, vija 10, vija 12, and vija 31, whose molecular weights were determined (Fig. 3A). This result matched with our sequences obtained from the transcriptome sequencing described above, and the primary structure of the cyclotides was deduced (Fig. 3A). Figures 2B and S2 showed the chromatogram graph and peak positions of these cyclotides. The figure (Fig. S3) contains the chromatogram and mass spectrum of all cyclotides.

To validate the primary sequence of cyclotide, enzymatic cleavage and tandem mass spectrometry were employed. Trypsin was utilized for the enzymatic cleavage of reduced alkylated cyclotides. Specifically, vija 10, known to contain two lysine residues, was cleaved into two fragments. The first fragment, comprising four amino acids (VCYK), spanned from the 27th to the 30th position, with a small 569.02 Da fragment exhibiting a 1 + charge. The second, a larger 987.93 Da fragment with a 3 + charge, encompassed amino acids from the 31st to the 26th position, represented by NGSIPCGESCVFIPCISALLGCSCSSK (Fig. 3B). Subsequently, ESI–MS/MS was employed to analyze the sequence of the digested peptides, with results depicted in the (Fig. 3C,D). Notably, all fragmented b and y ions were observed in the short peptide, while some b and y ion peaks were identified in the long peptides. This outcome reinforces the accuracy of the primary structural sequence of vija 10. Further analysis was conducted on viba 11, vija 12, and cycloviolacin O2 (cy O2) using tandem mass spectrometry (Table S2 and Figs. S4-6). Additional cyclic peptides extrapolated from the transcriptome data remain unidentified, potentially attributed to variations in the expression abundance of these cyclotides. Enhanced sensitivity and advanced mass spectrometry techniques are imperative for precise analytical identification.

### Cyclotide antimicrobial activity analysis

Cycloviolacin O2, known for its antimicrobial, hemolytic, and insecticidal activities, has been more extensively investigated^4,20,36–38^. Viba 11, originally discovered in *Viola baoshanensis*^39^ and found in other plants such as *Viola tricolor*^40^, *Viola betonicifolia*^3^, and *Viola philippica*^41^, lacks biological activity studies. These two cyclotides and vija 10 are high in content and easy to purify, and cycloviolacin O2 can also be used as a positive control to conduct activity study. In addition, few vija12 and 31 were extracted for antibacterial experiments. All purified cyclotides underwent antibacterial testing against Gram-positive bacterium *Staphylococcus aureus* (CMCC(B)26003-2-10) and *Bacillus subtilis* (CMCC(B)63501), Gram-negative bacteria, including *Acinetobacter baumannii* (ATCC19606), *Escherichia coli* (CMCC(B)44102-2-6), and *Salmonella paratyphi* (CMCC(B)50094-2-9). Notably, all cyclotides exhibited inhibitory activity against *A. baumannii*. The minimum inhibitory concentrations (MIC) for the tested cyclotides—viba 11, cycloviolacin O2, vija 10, vija 12, and vija 31 against *A. baumannii* were 10.8 μM, 4.2 μM, 57 μM, 14.9 μM, and 14.9 μM, respectively. In addition, the MICs of viba 11, cycloviolacin O2 and vija 10 against *B. subtilis* were respectively 5.4 μM, 2.1 μM and 14.3 μM (Table 1). Of these, cycloviolacin O2 demonstrated the highest antibacterial activity against *A. baumannii and B. subtilis*. Based on research findings, it has been demonstrated that the antimicrobial activity of cycloviolacin O2 is reduced when chemical masking of the charged Glu and Lys residues in cycloviolacin O2^42^. Therefore, it is hypothesized that the lower antimicrobial activity of vija 10 may be attributed to the presence of only one Lys residue in loop 5.

**Table 1:** Antimicrobial Effects of 5 Cyclotides (MIC/μM).

	viba 11	cycloviolacin O2	vija 10	vija 12	vija 31	Kanamycin sulfate
*Acinetobacter baumannii*	10.8	4.2	57	14.9	14.9	8
*Staphylococcus aureus*	> 21.6	> 16.8	> 28.5	ND*	ND	ND
*Escherichia Coli*	> 21.6	> 16.8	> 28.5	ND	ND	8
*Salmonella paratyphi*	> 21.6	> 16.8	> 28.5	ND	ND	ND
*Bacillus subtilis*	5.4	2.1	14.3	ND	ND	4

ND* Indicates not determined.

### Time killing kinetics

**Figure 4:**
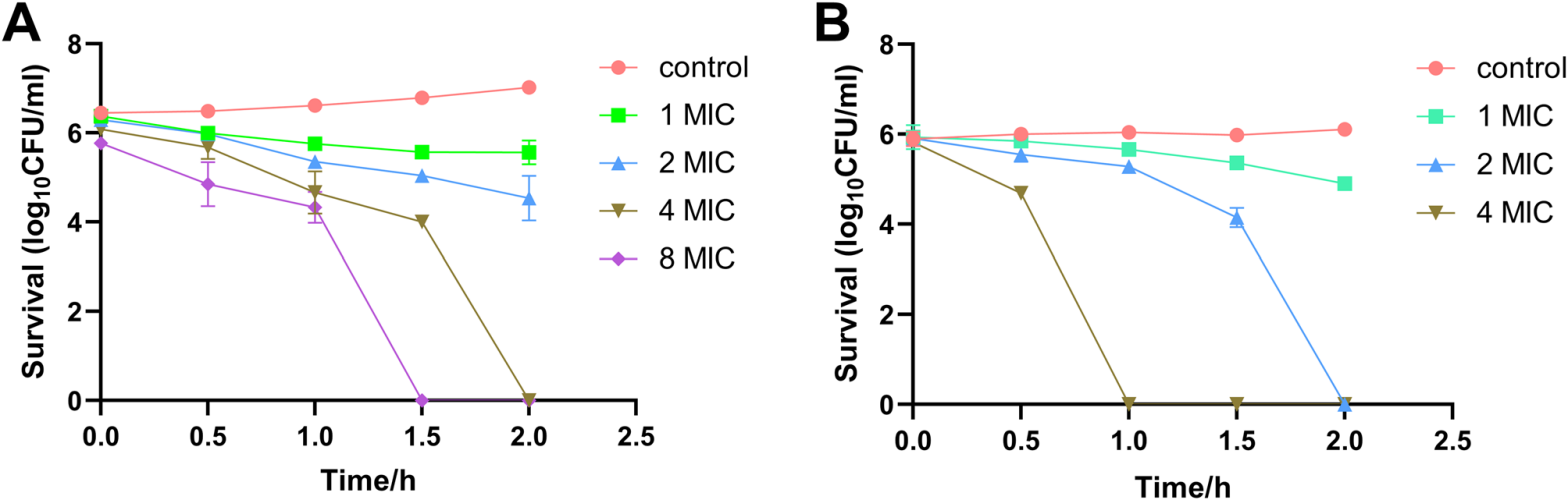
(**A**) The *Acinetobacter baumannii* time killing kinetics of cycloviolacin O2 at concentration of 1 MIC, 2 MIC, 4 MIC and 8 MIC at 37 °C. (**B**) The *Bacillus subtilis* time killing kinetics of vija 10 at concentration of 1 MIC, 2 MIC, 4 MIC at 37 °C. Each test was performed three times at least.

In this study, we investigated the time-kill kinetics characteristics of plant cyclotides against bacteria. We selected cycloviolacin O2, which exhibits the most potent antibacterial activity against *A. baumannii*, and conducted assays at different concentrations (1 MIC, 2 MIC, 4 MIC, and 8 MIC). According to the results (Fig. 4A), it was observed that after 1.5 h of treatment, cycloviolacin O2 at 8 MIC completely eradicated *A. baumannii*, while at 2 h, 4 MIC of cycloviolacin O2 also completely eradicated *A. baumannii*. It is worth noting that due to experimental constraints, there was a slight discrepancy of a few minutes between theoretical 0 h and actual 0 h. However, at the actual 0-h mark, the number of *A. baumannii* under 8 MIC condition was significantly lower than the negative control, indicating a rapid and effective bactericidal effect under the 8 MIC condition. Furthermore, we also conducted time-kill kinetics studies using vija 10 against *B. subtilis*. The results showed that vija 10 at 4 MIC effectively killed *B. subtilis* in the solution after 1 h of treatment, while 2 MIC of vija 10 completely eradicated *B. subtilis* after 2 h of treatment (Fig. 4B). These findings further underscore the potential application value of cyclotides in antibacterial activity, providing valuable insights for the future development of novel drugs with highly efficient antibacterial properties.

### Study of antimicrobial mechanism

**Figure 5:**
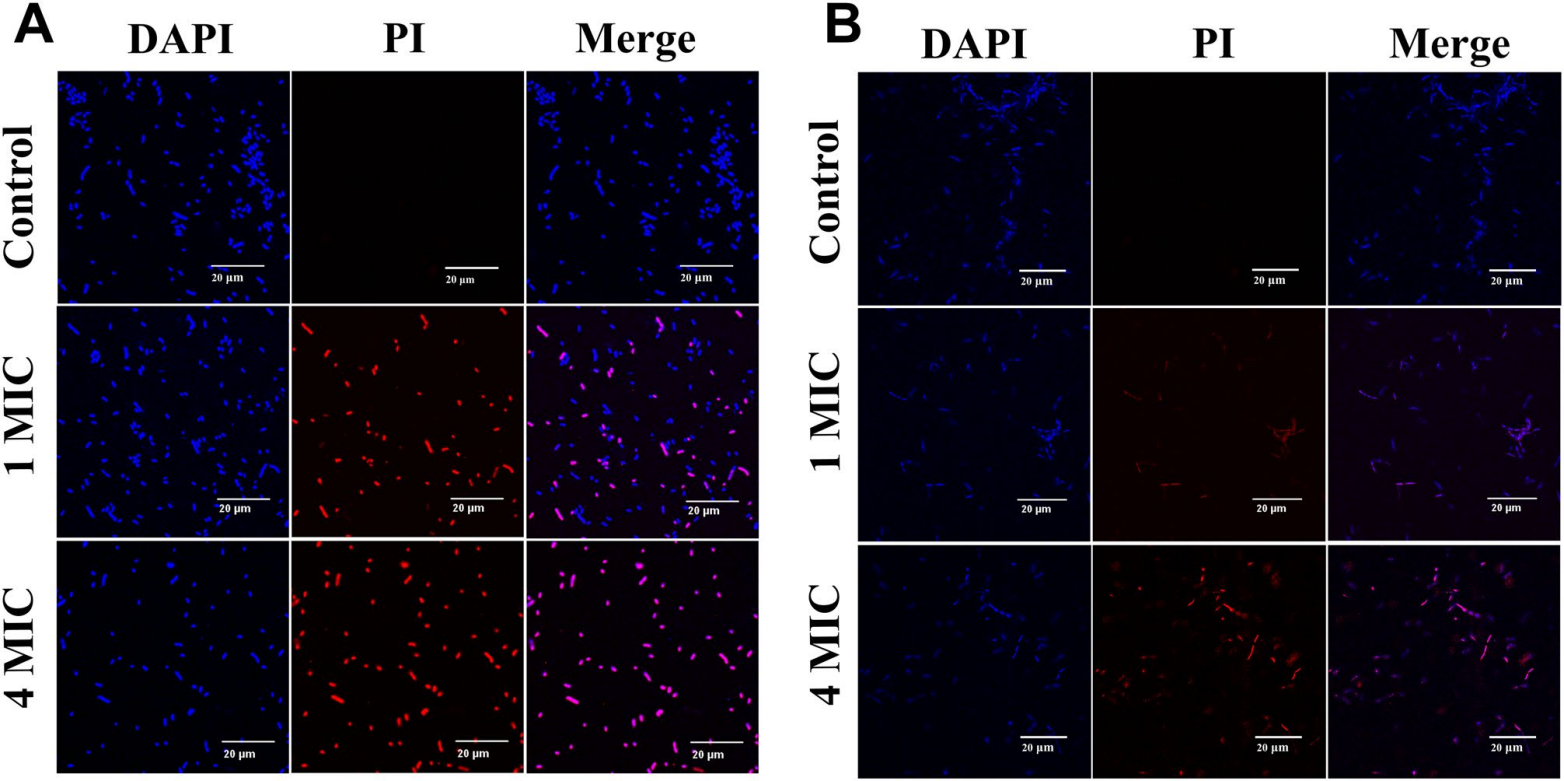
Study on the antimicrobial mechanism of cyclotides. (**A**) Incubation of *Acinetobacter baumannii* with sterile water, 1 MIC and 4 MIC of viba 11. (**B**) Incubation of *Bacillus subtilis* with sterile water, 1 MIC and 4 MIC of vija 10. All samples were stained with DAPI and PI. The negative control group PI is not stained and the membrane is intact, while the membrane treated with cyclotides was stained and the membrane are destroyed. Scale bar: 20 μm. Magnification: 600×.

It is found that the interaction of cyclotide with the membrane is the main reason for its biological activity^43,44^. Many naturally occurring antimicrobial peptides (AMPs) are cationic. Cationic AMPs (CAMPs) exert bactericidal activity by mechanisms featuring cell membrane disruption^45^. Cyclotides are likely to kill germ by disrupting their membranes. Laser scanning confocal microscopy can be used to study the ability of cyclotides to destroy bacterial membrane. 4′, 6-diaminyl-2-phenylindole (DAPI) is a blue fluorescent dye that can penetrate membranes and stain both living and dead strains, while propyl iodide (PI) stains only strains with damaged membranes, glowing red^46,47^. In this study, the bracelet cyclotide family members viba 11 and vija 10 interacted with *A. baumannii* and *B. subtilis* in their logarithmic phase at 37 °C for 1–2 h. After the addition of DAPI and PI fluorescent dyes, the samples were incubated in a dark room at room temperature for 15 min for staining^48^. The stained samples were then centrifuged to concentrate, resuspended, and applied to a slide for observation using a confocal laser scanning microscope (FLUOVIEW FV3000, Olympus). As shown in Fig. 5, sterile water solution without peptides served as a negative control, producing almost no red fluorescence, indicating intact bacterial strains with undamaged membranes. At 1 MIC, the cyclotides caused damage to the membrane integrity of both *A. baumannii* and *B. subtilis*, releasing partial red fluorescence. With increasing concentration, the blue and red fluorescence gradually overlapped. At 4 MIC, the staining of the nucleic acid dyes DAPI and PI was highly consistent, indicating that the majority of the cell membranes were ruptured, leading to bacterial death. However, the magnification is not sufficient to detect further details of membrane disruption. Therefore, further exploration can be conducted using methods such as scanning electron microscopy (SEM) to study the antibacterial mechanism in depth.

### Cytotoxicity activity

Five types of cells, namely A549 (human non-small cell lung cancer cells), T24 (human bladder cancer cells), 4T1 (mouse breast cancer cells), 7402 (human hepatocellular carcinoma cells), and THLE-3 (human hepatocytes cells), are selected for evaluating the cytotoxicity of viba 11, cycloviolacin O2, and vija 10 using the MTT (3-(4,5-dimethylthiazol-2-yl)-2,5-diphenyltetrazolium bromide) reagent. The mean of IC_50_ and SD of cyclotides for various cells are showed (Table 2).

**Table 2:** Toxicity of cyclotides in *Viola japonica* against mammalian cells (IC_50_/μM, mean ±SD).

Name	viba 11	cycloviolacin O2	vija 10
A549	0.195 ± 0.09	0.149 ± 0.03	0.733 ± 0.20
T24	0.724 ± 0.17	1.689 ± 0.16	3.276 ± 0.15
4T1	0.285 ± 0.01	0.162 ± 0.01	0.244 ± 0.02
7402	1.696 ± 0.05	1.332 ± 0.01	2.175 ± 0.06
THLE-3	1.609 ± 0.01	1.209 ± 0.05	2.086 ± 0.06

A549: human non-small cell lung cancer cells, T24: human bladder cancer cells, 4T1: mouse breast cancer cells, 7402: human hepatocellular carcinoma cells, THLE-3: human hepatocytes cells.

**Figure 6:**
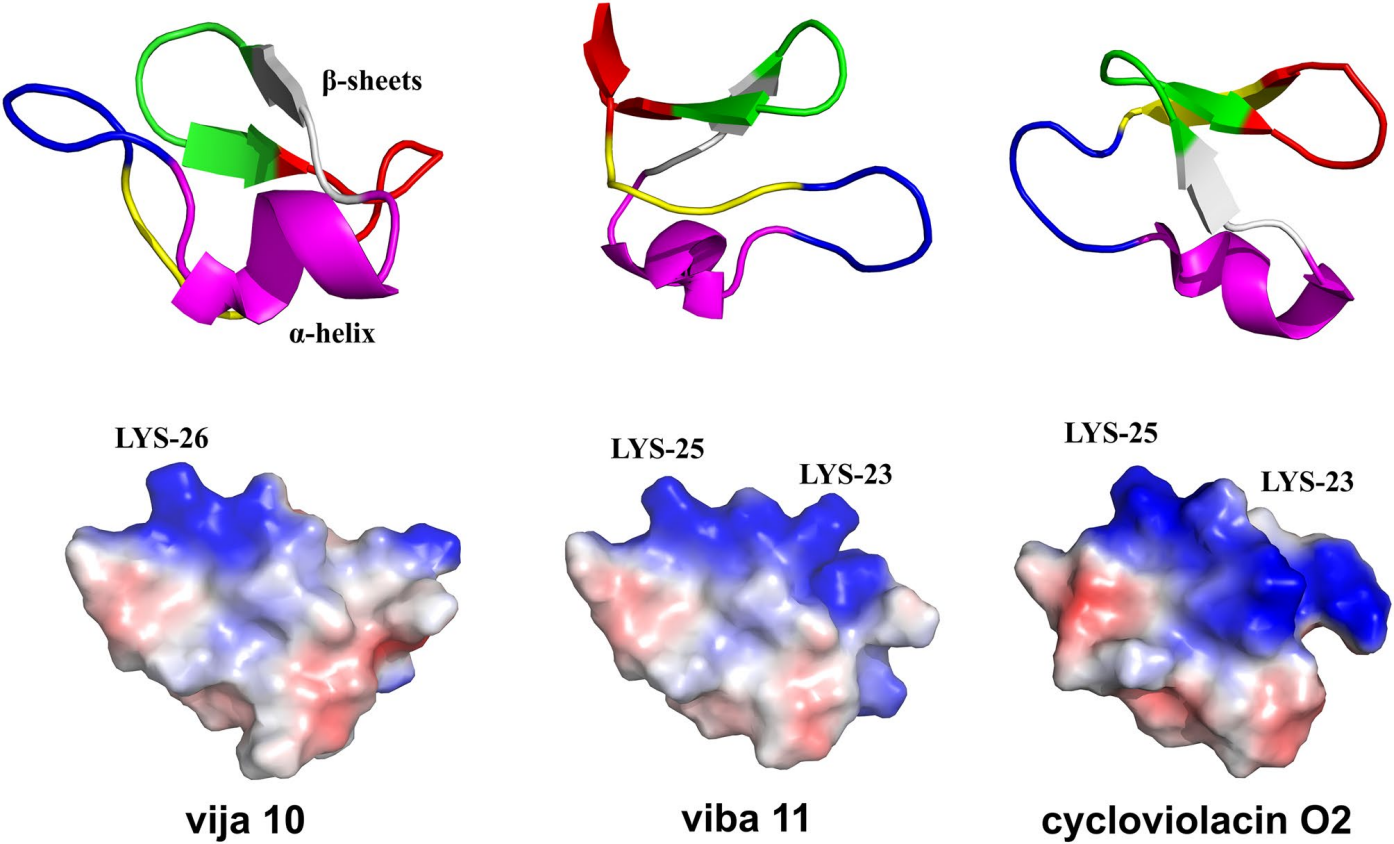
Structure prediction of cyclotide vija 10 and viba 11 using Alphafold 2 and cycloviolacin O2 (PDB:7RMQ) which all contain a short α-helix in loop 3 and antiparallel β-sheets with loops 4 and 5 and their surfaces, with electric potential depicted in red (indicating negative charge) and blue (indicating positive charge). In the structures of the cyclotide, each loop region is marked with a different color, loop 1: yellow; loop 2: blue; loop 3: purple; loop 4: gray; loop 5: green; loop 6: Red.

The IC_50_ values of these three cyclotides for different cancer cells range from 0.1 to 3.5 μM, which is comparable to the toxicity reported in previous studies concerning cyclotides^4,19,20^. The cytotoxicity moderately varies across different cancer cells, with cycloviolacin O2 displaying the most potent activity against A549, 4T1, 7402, and THLE-3 cells, with IC_50_ values of 0.149 μM, 0.162 μM, 1.332 μM, and 1.209 μM, respectively. Cycloviolacin O2, being extensively studied cyclotide, exhibits a broad range of cytotoxicity. viba 11 demonstrates the highest activity against T24 cells with an IC_50_ value of 0.724 μM, possibly due to its similar composition and structure to cycloviolacin O2. On the other hand, vija 10, despite having a relatively higher IC_50_ value, still falls within the μM range. As shown in Figs. 3A and 6, the sequences of viba 11 and cycloviolacin O2 are similar, differing only in the first amino acid in loop 3, glycine for viba 11 and serine for cycloviolacin O2. The cyclotide vija 10, belonging to the bracelet cyclotide subfamily along with them, differs by containing fewer positively charged amino acids in loop 5 and more hydrophobic amino acid. Possibly the distribution of charged and hydrophobic residues determines the potency of cytotoxicity^49^.

### Structure prediction

In this study, all recently identified cyclotides are classified within the bracelet and Möbius subfamilies. Specifically, viba 11 and vija 10, in addition to cycloviolcin O2, are categorized under the bracelet subfamily. Subsequently, Alphafold 2 was employed to forecast the three-dimensional and secondary structures of viba 11 and vija 10^50^. The Predicted Local Distance Difference Test (pLDDT) is a metric for assessing the confidence level in predicting the accuracy of protein structures. pLDDT scores range from 0 to 100, with higher scores indicating greater confidence in the predicted structure at the corresponding position. Typically, scores exceeding 90 indicate a very high level of confidence. In this study, the pLDDT scores for vija 10 and viba 11 are 91.2 and 92, respectively, indicating a high level of confidence in the obtained structures. The anticipated outcomes, together with the structural data of cycloviolacin O2 (PDB: 7RMQ)^51^, were processed utilizing Pymol software, revealing the presence of short α-helices in loop 3, and antiparallel β-sheets in loops 4 and 5. These structural attributes are characteristic of conventional bracelet cyclotide configurations (refer to Fig. 6). This observation also suggests a degree of homogeneity in their biological functionalities. Analysis of the electrostatic potential maps of the cyclotides indicates notable distinctions. Cycloviolacin O2 exhibits a more extensive blue-colored area and higher electrostatic potential, whereas vija 10 displays a smaller blue-colored area and lower electrostatic potential. Considering the antimicrobial activity and cytotoxicity findings, it is plausible that the heightened activity of cycloviolacin O2 correlates with its elevated electrostatic potential.

## Conclusion

In summary, this research focuses on *V. japonica* and utilizes transcriptome analysis to identify a total of 61 cyclotides, consisting of 25 previously reported ones and 36 newly discovered ones. The cyclotides viba 11, cycloviolacin O2, and vija 10 are acquired during the extraction process and are subsequently tested for antibacterial activity and cytotoxicity. All of them show anti-*A. baumannii* and *B. subtilis* activity, cytotoxicity. Antibacterial peptides are often degraded by proteases, which limits their clinical application to local applications. Cyclotides, distinguished by their stable structure and unique cysteine configuration, are less prone to degradation and hold promise as a novel class of antibacterial agents. Furthermore, through the use of fluorescent staining, it is observed that the cyclotides induce bacterial membrane disruption*.* These findings provide substantial evidence of the potent activity possessed by cyclotides. Moreover, they contribute to the expanding diversity of cyclotides and enhance their potential value in various applications. In the future, the subsequent research directions can involve the investigation of cytotoxic mechanisms and the chemical synthesis of cyclotides, particularly within the bracelet cyclotide subfamily, to enable the large-scale application of cyclotides. The oxidation-folding process for the bracelet cyclotide subfamily is relatively challenging, as it tends to produce non-native folded variants. Therefore, the development of cyclotides should not solely rely on extraction. Improving cyclotide synthesis methods, especially the oxidation-folding process, is essential for the advancement of cyclotides.

## Experimental

### Plant material

The plant was collected with permission in Qingyuan City, Guangdong Province, China, from July to September, complying with the *International Plant Protection Convention* and *Regulations of the People's Republic of China on the Protection of Wild Plants*. Dr. Wang Lei from the School of Forestry, Guangxi University, China, identified the plant materials used for research as *V. japonica*. A voucher specimen was deposited in the Guangxi Key Laboratory of Special Biomedicine at Guangxi University. The extraction of cyclotides follows the published methodology^20^.One kilogram of fresh *V. japonica* was washed and cut into small pieces, and then immersed in a 50% acetonitrile water solution containing 1% formic acid shake overnight for extraction. The crude extract was filtered and centrifuged at 9000 rpm for 10 min at 4 °C, and the supernatant was collected and freeze-dried.

### Transcriptome analysis

The collected *V. japonica* was separated based on different parts, including roots, stems and leaves. Each part was then cooled and ground in liquid nitrogen for RNA extraction, followed by preliminary concentration and purity detection using Nanodrop. Integrity and concentration accuracy of the RNA were checked using the Agilent 4200 Bioanalyzer, and qualified RNA samples were subjected to library construction. The NEBNext^®^ Ultra™ RNA Library Prep Kit for Illumina^®^ (NEB, USA) was used for constructing eukaryotic transcriptome libraries. The qualified libraries were sequenced on the Illumina Novaseq6000 (Illumina, USA) high-throughput sequencing platform using PE150 (Pair-End 150) sequencing strategy, with a minimum data volume of 6 Gb per sample. Three sets of data were separately arranged for non-reference transcriptome assembly. Analysis of transcriptome sequencing data without a reference genome was performed using the Trinity transcriptome sequence assembly strategy. Through a three-step process of Inchworm, Chrysalis and Butterfly, high-throughput sequencing data was assembled into transcript sequences.

A. Inchworm: Assemble reads into contigs using a kmer-based assembly strategy;

B. Chrysalis: Cluster contig sequences, define components, and align reads back to components to validate their correctness;

C. Butterfly: Assemble components into putative transcripts using a De Bruijn graph-based assembly strategy.

Screen cyclotides from their characteristics, such as ending with D or N, containing 6 cysteine residues, and having SI, SL, AL at the CTR beginning and range of 25 to 40 amino acids, simultaneously, using known cyclotide sequences such as kalata B1 and cycloviolacin O2 et al. as templates, a BLAST search was performed.

### SPE purification

The freeze-dried crude extract was re-suspended in a 1% TFA water solution, and an SPE column (Waters Sep-pak C18) was prepared by pre-activation and equilibration with methanol and a water solution containing 1% TFA. The crude extract was loaded onto the SPE column, and impurities were washed away with a 10% acetonitrile water solution containing 1% TFA. The elution was carried out sequentially with 20%, 30%, 50%, and 80% acetonitrile water solutions containing 1% TFA, and the fractions were collected.

### RP-HPLC purification

Separate the collected solution using a preparative liquid chromatography system (Waters Prep 150 preparative liquid chromatography system). Filter and dilute the collected solution, and inject it into the preparative liquid chromatography column (SunFire prep C18 OBD) for separation. The mobile phase A is a water solution containing 0.1% TFA, and the mobile phase B is a 90% acetonitrile–water solution. The flow rate is set at 12 ml/min, and the gradient condition is set to increase mobile phase B from 5 to 70% within 40 min. The absorption wavelength is set at 214 nm.

### Mass spectrometry determination

The purified sample was analyzed by Waters ACQUITY TQD tandem quadrupole mass spectrometer for molecular weight determination through ion scanning. The mobile phase A was a 0.05% formic acid aqueous solution, and the mobile phase B was a 100% acetonitrile solution. The parameters were set as follows: cone voltage 45 V, cone gas flow rate 1000 L/Hr, capillary voltage 1.2 kV, and scan range 400–2000 Da.

### Alkane reduction and enzyme degradation.

Due to the presence of 3 pairs of disulfide bonds and 6 cysteine residues in natural cyclotides, they were reduced using dithiothreitol under alkaline conditions to release the thiol groups. Afterwards, iodoacetamide was added to react with the released thiol groups to prevent the reformation of disulfide bonds. The cyclotide mass was determined using ESI–MS, and the mass of the reduced and alkylated cyclotides was theoretically increased by 348 Da compared to that of the natural cyclotides. Trypsin was added to the reduced and alkylated cyclotides for 1 h at 37 °C, and the fragment information was determined using tandem mass spectrometry sequencing.

### Quantification of cyclotides

The concentration of cyclotides can be determined by UV absorption at 280 nm, mainly relying on the content of tryptophan and tyrosine in cyclotides^52^. Protein concentration can be calculated using the Lambert–Beer law A = εcl, where A is the absorbance at 280 nm, ε is the extinction coefficient of cyclotides, l is the path length in cm, and c is the molar concentration. The extinction coefficient of cyclotides can be calculated using the following formula:

$$\varepsilon_{{{28}0{\text{ nm}}}} = \left( {{\text{n}}_{{{\text{w}}*}} {55}00} \right) + \left( {{\text{n}}_{{{\text{Y}}*}} {149}0} \right) + \left( {{\text{n}}_{{{\text{s}} - {\text{s}}*}} {125}} \right)$$

n_W_ represents the number of tryptophan residues, n_Y_ represents the number of tyrosine residues, and n_S-S_ represents the number of disulfide bonds. All cyclotides contain three rings^53^.

### Antimicrobial activity analysis

Bacterial fluids are cultured overnight at 37 °C. The next day, the bacterial liquids are diluted to a concentration of 10^6^ CFU/ml. Prepare different concentrations of cyclotide drugs and kanamycin sulfate in advance, and sequentially add the drugs and bacterial liquid to the 96-well plate. Negative controls consisted of sterile water and bacterial liquid. The plate is cultured overnight at 37 °C and observe whether the solution remains clear to see the growth of the bacterial suspension. Each test was performed three times at least.

### Laser confocal sample preparation

Take 50 μL of *A. baumannii* and *B. subtilis* (10^8^ CFU/mL), and add 50 μL of sterile water, different concentrations of cyclotides viba 11 and vija 10 to achieve final concentrations of 1 MIC and 4 MIC respectively. Incubate at 37 °C for 1–2 h. Subsequently, add 4,6-diamino-2-phenylindole (DAPI) and propidium iodide (PI) dyes at 20 μg/mL each, and incubate in the dark for 15 min. Centrifuge at 5000 rpm for 3 min to concentrate, discard the majority of the supernatant, and resuspend the bacteria in the remaining small volume of liquid. Mix well and take 10 μL of the stained sample onto a glass slide, gently place a cover slip on top with forceps, avoiding the formation of bubbles during the pressing process. After sealing the cover slip, check with a laser confocal microscope, and the observation was conducted using an objective lens with the maximum magnification of 60 × . The total magnification = 10 × (eyepiece magnification) * 60 × (objective magnification) = 600 × .

### Time killing kinetics

Incubate *A. baumannii* and *B. subtilis* in fresh nutrient LB broth at 37 °C until logarithmic growth stage, then dilute to approximately 10^6^ CFU/mL, and incubate with cycloviolacin O2 at concentrations of 1 MIC, 2 MIC, 4 MIC, 8 MIC with *A. baumannii* and vija 10 at concentrations of 1 MIC, 2 MIC, 4 MIC with *B. subtilis* respectively. Taking the mixture at time points of 0 h, 0.5 h, 1 h, 1.5 h and 2 h, dilute and coat it to solid medium for 24 h. Count the number of colonies on solid culture medium. Each test was performed three times at least. Plotting a line graph with time on the x-axis and bacterial survival on the y-axis using GraphPad Prism 8. Statistical analysis of bacterial viability at different time points to determine the bactericidal and bacteriostatic effects of cyclotides.

### Cytotoxicity assay

To determine cell toxicity using MTT assay, cells are seeded in a 96-well plate and cultured for 24 h. After that, different concentrations of cyclotides are added to the cells and incubated for 48 h. MTT is then added to react with the cells, and after 4 h, the reaction is terminated by discarding the supernatant and adding dimethyl sulfoxide for resuspension. The absorbance at 570 nm is measured after 10 min of shaking at room temperature. Each test was performed three times at least. The data were analyzed using GraphPad Prism 8. During this process, the drug concentrations were transformed into logarithmic form with a base of 10 and plotted on the x-axis. Cell viability (%) = [A _(cyclotides)_ − A _(blank)_]/[A _(control)_ − A _(blank)_] × 100, which was represented on the y-axis. Using this method, we determined the IC_50_ value for each experiment, with each experiment repeated at least three times and the mean value calculated. The error was represented by the standard deviation of the IC_50_ obtained from three repetitions.

### Structure prediction of cyclotides

Structural prediction of the cyclotides viba 11 and vija 10 was conducted utilizing the Colab server (https://colab.research.google.com/github/sokrypton/ColabFold/blob/main/AlphaFold2.ipynb)^50^. The cyclotide sequences and file name were inputted, default parameters were employed, and the process was executed to procure results. This server employs a marginally simplified iteration of AlphaFold v2.0, initiating peptide structure predictions directly from their sequences without reference to pre-existing structural templates. Structural diagrams and electrostatic potential maps of the predicted cyclotides as well as cycloviolacin O2 (PDB:7RMQ)^51^ were generated using Pymol.

### Supplementary Information

Supplementary Information.

## Data Availability

The raw sequence data reported in this paper have been deposited in the Genome Sequence Archive1 in the National Genomics Data Center, China National Center for Bioinformation (GSA: CRA014480) that are publicly accessible at https://ngdc.cncb.ac.cn/gsa, and some data is provided in the supplementary information files.
