# Supplementary material for: Characterization and evaluation of cytotoxic and antimicrobial activities of cyclotides from Viola japonica
Source: Sci Rep. 2024 Apr 28;14:9733. doi: 10.1038/s41598-024-60246-9 (PMC11056381; doi:10.1038/s41598-024-60246-9)
Supplement: Supplementary file 1 — Supplementary Information. [file 41598_2024_60246_MOESM1_ESM.rtf]

Characterization and Evaluation of Cytotoxic and Antimicrobial Activities of Cyclotides from Viola japonica


Yuanyuan Lian1, Xue Tang1, Gehui Hu1, Chenfang Miao3, Yunfei Cui1, Dongting Zhangsun1,2, Yong Wu1* and Sulan Luo1, 2*
1Guangxi Key Laboratory of Special Biomedicine; School of Medicine, Guangxi University, Nanning, China
2Key Laboratory of Tropical Biological Resources of Ministry of Education, Hainan University, Haikou, China
3Department of Pharmacy, The 900th Hospital of Joint Logistics Team of the PLA, Fuzhou General Clinical Medical College of Fujian Medical University, Fuzhou, China
*Email: wuyong@gxu.edu.cn, sulan2021@gxu.edu.cn


Cyclotide precursor sequences from Viola japonica through transcriptome sequencing (cDNA&AA)
>vija1 
AGCATCACCATGACCAAAACCATCGTCTCAAACCCAATCCTTGAAGAAGCACTGGTTGCCTATTCCAACCACAAACTTGGAGGTGGCACCATTTTCGACTGTGGTGAAACTTGCTTTCTCGGTAAATGCTACACTCCTCATTGTCTTTGCGGCAAATATAAATTTTGCTATGGCCAAGATTCTCTAGAAAAAAACAGCAACAAGGCAGGGAAAGAGGCACTCGTTGATTTTGCATCTTTCGAAACGAAAGATTTCATCACTCGTGGCGCATACGAAAATCTTGTCAAGAGCGGTGCCATTGAGGGTAGCATCACCATGACCAAAACCATCGTCTCAAACCCAATCCTTGAAGAAGCACTGGTTGCCCATTCCAACCAAAAACTTGGAGGTGGTACCGGTACCATTTTCGACTGTGGTGAATCTTGCTTTTTGGGTAAATGCTACACTCCTCATTGTAGTTGCGGCGAGTATTTTTTCTGCTATGGCACAGATTCTCTAGAAAGAAACAGCAACAAGGCAGGGGAAGAGGCACTCGTTGCTTTAGCCAGGAAAGGTCTTGCAGCTGCTCTTGCTGGTCTTGCTAAT

SITMTKTIVSNPILEEALVAYSNHKLGGGTIFDCGETCFLGKCYTPHCLCGKYKFCYGQDSLEKNSNKAGKEALVDFASFETKDFITRGAYENLVKSGAIEGSITMTKTIVSNPILEEALVAHSNQKLGGGTGTIFDCGESCFLGKCYTPHCSCGEYFFCYGTDSLERNSNKAGEEALVALARKGLAAALAGLAN
>vija2
TTAATAAGAGCTACAGCTAAAACCATAACAAATCAGTCAATCAATATTATTATGGAGAGCAACAAGAAGATGCAGCTTGTTGGGTTTGTGCTCCTGGCCGCCTTTGCGCTACCAGCACTTGTCACATCTTTCGAGAAAGATGTGATCTCTGCCAGTGCCATCCAGGCTGTCCTGGAGAAGAGGGGATTAAGCAAGCTGGAGGATGATCCTGTCTTGAGCGCTCTTGCTCGCACCAAAACCATAATCTCTAACCCTGTCATCGAAGAGGCTTTGCTTAATGGTGCCAGCCTGAAAGCAGGTAATTACATCCCCTGCGCCGAGAGCTGCGTGTATATTCCATGCACAGTCACAGCATACGTATTTGGGTGCTCATGCAAGGACAAAGTTTGCTGGAACTCTCTCGAAACCAAGTAC

LIRATAKTITNQSINIIMESNKKMQLVGFVLLAAFALPALVTSFEKDVISASAIQAVLEKRGLSKLEDDPVLSALARTKTIISNPVIEEALLNGASLKAGNYIPCAESCVYIPCTVTAYVFGCSCKDKVCWNSLETKY
>vija3 
ATGAAGATGGTGGTCGGTAACGTGCTTGTGGTGGCTGCTGCGGCCTTTGCTCTGATCCCTGCATCCTTTACAGTGGAAGATATCATCACTCACGATCCGTACGGGAATCTTGTTACAAGCAGAGCCATTGATGGCATTATCATGACCAAAACCGTCATCTCAAACCCAATCTTTGAAGAAGCACTGCTTACCTATTCCATCAACAAACTTGGTGGTCGTGCCGTTTGTGGGGAAACTTGCTTTACGGGTATATGCTACACTCCTATTTGTGTTTGCGGCAAATGGGACTTATGCCGCATGAATTCTATACAGCAAGTT

MKMVVGNVLVVAAAAFALIPASFTVEDIITHDPYGNLVTSRAIDGIIMTKTVISNPIFEEALLTYSINKLGGRAVCGETCFTGICYTPICVCGKWDLCRMNSIQQV
>vija4
TTTGCAGCCTTTGCTCTCCCAGCTGCCTTTGCAAGTAGAAACTCTTTCGATGAAAAGAACAGTCTTATTCTCGAGGAGGCACTCGTTGCGTTCGCCAAAAGAGACGATGTCAATGGGCTCAACTGCGGTGAGACTTGTTGGGGGTTTACTTGCGACCGCCCCGATTGCTCTTGTGGGTTGACGTATCCTTTTTGCGCCAAAAATTCTCTCGCCATG

FAAFALPAAFASRNSFDEKNSLILEEALVAFAKRDDVNGLNCGETCWGFTCDRPDCSCGLTYPFCAKNSLAM
>vija5
CTCTCAAACATCATGTTAGAGGAAGATGCCATGAGTGCTCTTATCAAGAGCAAGACCGTCATCTCCAACCCAGTTATCGAAGAGGCACTCCTCAAGAACAGTAACAGTCTTCACGGCATCCCTTGTGCTGAAAGTTGTGTATTTCTTCCATGCGTGACTGTTGTTCTTGGGTGTTCTTGCAAGGACAAAGTTTGCTACAACTCTCTTGATATA

LSNIMLEEDAMSALIKSKTVISNPVIEEALLKNSNSLHGIPCAESCVFLPCVTVVLGCSCKDKVCYNSLDI
>vija6
AGCATCACCATGACCAAAACCATCGTCTCAAACCCAATCCTTGAAGAAGCACTGGTTGCCTATTCCAACCACAAACTTGGAGGTGGCACCATTTTCGACTGTGGTGAAACTTGCTTTCTCGGTAAATGCTACACTCCTCATTGTCTTTGCGGCAAATATAAATTTTGCTATGGCCAAGATTCTCTAGAAAAAAACAGCAACAAGGCAGGGAAAGAGGCACTCGTTGATTTTGCATCTTTCGAAACGAAAGATTTCATCACTCGTGGCGCATACGAAAATCTTGTCAAGAGCGGTGCCATTGAGGGTAGCATCACCATGACCAAAACCATCGTCTCAAACCCAATCCTTGAAGAAGCACTGGTTGCCCATTCCAACCAAAAACTTGGAGGTGGTACCGGTACCATTTTCGACTGTGGTGAATCTTGCTTTTTGGGTAAATGCTACACTCCTCATTGTAGTTGCGGCGAGTATTTTTTCTGCTATGGCACAGATTCTCTAGAAAGAAACAGCAACAAGGCAGGGGAAGAGGCACTCGTTGCTTTAGCCAGGAAAGGTCTTGCAGCTGCTCTTGCTGGTCTTGCTAAT

SITMTKTIVSNPILEEALVAYSNHKLGGGTIFDCGETCFLGKCYTPHCLCGKYKFCYGQDSLEKNSNKAGKEALVDFASFETKDFITRGAYENLVKSGAIEGSITMTKTIVSNPILEEALVAHSNQKLGGGTGTIFDCGESCFLGKCYTPHCSCGEYFFCYGTDSLERNSNKAGEEALVALARKGLAAALAGLAN
>vija7
AAGAATATGGTTGTTGGGCTCTTGCTCATTGCTACCTTTGCTCTTCCAGCCCTTGCAACAAGCTTTGAGAAAGATTTCATCACACATGAAACCATCCAGACCATTGTTAAGAAGGTCGGTCTCAATTCCAACGGGATGCTAGATGAGCAAACCAGCCTTGAGAAAGATGTGATCACACATGAAACTGTCCAGGCAATTCTTATGAAGGTCGGTCGTTCTAATTTAGATGGGATGCTAGATGAGCAAACTATCAGTGCTCTCACCGGCAAGGTTATCATCTCCAACCCACTTTTTGAAGAGGCACTCTTCAAGCAGTCCTTTCCTAACGGACCCATTGATTGCAGAGAAACTTGTGTTTGGACTCCTTGCAAAAGCGTCCTTATGAATTGTCGTTGCAGGCAAGGTATTTGCTTCAGGAACTCTCTTGCT

KNMVVGLLLIATFALPALATSFEKDFITHETIQTIVKKVGLNSNGMLDEQTSLEKDVITHETVQAILMKVGRSNLDGMLDEQTISALTGKVIISNPLFEEALFKQSFPNGPIDCRETCVWTPCKSVLMNCRCRQGICFRNSLA
>vija8
GTCATGAATGCTATTATCAAGACCAAGACCGTCATCTCCAACCCACTTATCGAAGAGGCACTCCTGAAGAACAGTAACGGTCTTCACGGCGAATTTTGCGGGGAAACTTGTGTCGCGTTTCCATGCTTCAGTACTGCACATGGGTGTGGCTGCTATCAAATGGGGTGCGTCAAGAACTCTCTTGATATA

VMNAIIKTKTVISNPLIEEALLKNSNGLHGEFCGETCVAFPCFSTAHGCGCYQMGCVKNSLDI
>vija9
ATGAAGATGTTTATTGTGCTTGTGCTCTCTGCAGCTTTTGCTCTCCCTGCTGCCTTTGCAACTGCGCAAGATGTCATCACTCTCCAAGCTTACGAGGAGCTTCTCAAGAGTGGGGATGCTAATGGGATGACCAAAACTGTCATCTCAAGCCCTGTTCTTGAAGAGGCTCTCGTCTCTTACTCCAAGAACAAGCTCGGCGGTTTGCCTGTCTGCGGAGAGACTTGCACGTTGGGAACATGCTACACCGCTGGGTGCTCCTGCAGCTGGCCAGTCTGCACCAGAAACTCTCTTGAAAGCACCAAATCAGCAAACCCTCTCCTTGAAGAGGCGCTCACCGCGTTCGCCAAGAAAGGTCTTGGTGGTCTTCCCATATGCGGTGAGACTTGCGTCGGCGGAACATGCAACACCCCTGGG

MKMFIVLVLSAAFALPAAFATAQDVITLQAYEELLKSGDANGMTKTVISSPVLEEALVSYSKNKLGGLPVCGETCTLGTCYTAGCSCSWPVCTRNSLESTKSANPLLEEALTAFAKKGLGGLPICGETCVGGTCNTPG
>vija10
ATGGATGCCAGGAAAGTCTTTGTTGCCTTGGTTCTCATTGCAACCTTTGCACTTCCATCTTTTGCAACCTTCGAGAAAGATTTCATTACCCCAGAAACCGTTCGTGCTGTTCTCAGGGAGACCAACTCCAATGCCATGCCTTCAGAGCAAGCCATTAATGCCCTCACTGGCAAGACCCTCGTCTCAAACATAGTTGTCCAGGAGGCTCTCCTCAAGAACCTCGACAACGGTCTTAAAGGCAGCATCCCTTGCGGTGAAAGTTGTGTCTTTATTCCTTGCATCTCAGCCCTGCTTGGGTGTTCATGCAGCAGCAAAGTTTGCTACAAGAACTCCCTTGCCATG

MDARKVFVALVLIATFALPSFATFEKDFITPETVRAVLRETNSNAMPSEQAINALTGKTLVSNIVVQEALLKNLDNGLKGSIPCGESCVFIPCISALLGCSCSSKVCYKNSLAM
>vija11
CTTGCAACCTTTGAGAGAGATGTCATCACACCTGAAGCTATTCAGGCTATTCTTGATCAGAAGAAAACCAACCCCAACTCCAATTACATGCTCTCAGACTCGGAGGATGCCGTTAATGCCCTCACCGGCAAGATCGTCATCGCTGACAACCTCTTTCTTGAGGAGGCGCTCAGGATGATGAGGAGTAATAGTGTCAAGTTGGATGGAATCCACTGCGCGGAAACTTGTTTCTGGGGTACATGCCGCACTGCATACATTGGGTGTTCTTGCGAGAACAAAATTTGTTACAAGAACTCTCTTCTTGCCAAC

LATFERDVITPEAIQAILDQKKTNPNSNYMLSDSEDAVNALTGKIVIADNLFLEEALRMMRSNSVKLDGIHCAETCFWGTCRTAYIGCSCENKICYKNSLLAN
>vija12
ATGAAAGAAAGAAGCATGGATGCCCAAAAGATGAAGATGGTGATCGGTCTTGTGCTCGTCGCCACCGCGGCTTTTGCTCTGATGATCCCTGCAGCTTCTGCAGTGGACGATTTCATCACTCGTCGAGCATACGATAATCTTGTCAAGAGCGGTGCCATCAATGACATTCCAATCATGGCCAAAACCATCATCTCAAACCCAGTCCTTGAAGAAGCACTGCTTAATTATTACTCTAAGGACAAACTCGGCGGTAGCTACATTTCATGTGGGGAAACTTGTGTCAAGTTAAAATGCTACACTCCCGGGTGTACATGCACCTGGCCCGCATGCAAAAAAAATTCTCTAGACGACAGTACCACGGCTGATGAGGAGGCAGTATTCGCTGCTGCGTTTGCCAGGAAAGCTCTTGCAGGTCTTAATGCTAAT

MKERSMDAQKMKMVIGLVLVATAAFALMIPAASAVDDFITRRAYDNLVKSGAINDIPIMAKTIISNPVLEEALLNYYSKDKLGGSYISCGETCVKLKCYTPGCTCTWPACKKNSLDDSTTADEEAVFAAAFARKALAGLNAN
>vija13
GCTCTTACCAGTAAGATCCTCATCTCCAACCCGGTCATCGAAGAAGCACTCCTTAAGCACTCCAACTTTAATGCCCTCGGGGGCAGTGTTCCTTGCGGTGAAAGTTGTGTCTGGATTCCTTGCATTTCTAGCCTTGCTGGGTGCTCTTGCTCGAACAAAGTTTGCTACCTGAACTCTCTTGCCAAC

ALTSKILISNPVIEEALLKHSNFNALGGSVPCGESCVWIPCISSLAGCSCSNKVCYLNSLAN
>vija14
ATGGAGAACGACGCGATCGTGAATGTTATCGCCAACGTCAAGACCGTCATCTCAAATCCCGTGCTCGAAGAGGCGTTGTTTAAGACCAACCATGGTGTTAATGGCAAATCCTGCGGTGAAAGCTGTGTCTTTATTCCGTGCATCACCTCCGCAATTGGGTGTTATTGCAGTAGCAACGTTTGCTCCAGGAACTCTCTCCACAAC

MENDAIVNVIANVKTVISNPVLEEALFKTNHGVNGKSCGESCVFIPCITSAIGCYCSSNVCSRNSLHN
>vija15
CTTGAAGAGGCGCTCACCGCGTTCGCCAAGAAAGGTCTTGGTGGTCTTCCTACCTGCGGTGAGACTTGCTTCACCGGAGTATGCTACACTCCTGGGTGCCAGTGCGACTGGCCAATGTGCACAAAAAATGCTCTTGAGATTAAGAAACCCAACCATTTGCTCGAAGAGGCACTCGTTGCATTCGCCAAGAAAGGTAACCTCGGAGGTCTTCCCGTATGCGGCGAAACTTGCTTTGGTGGAACCTGTAACACCCCT

LEEALTAFAKKGLGGLPTCGETCFTGVCYTPGCQCDWPMCTKNALEIKKPNHLLEEALVAFAKKGNLGGLPVCGETCFGGTCNTP
>vija16
ATGGATTCCAAGATTGTGTTTGTTGCCCTTGTCCTCATTGCAACCTTTGCCCTTCCATCCCTTGCAACCTTTGAGAAAGATTTCATCACCACTGAAGCTGTTAGGGCTATCCTCAAGAAAACCAACTCCAATGCCATGCCCTCAGAGGATGTCATTATTGCCCTCACCGGCAAGACCCTCATCTCGAGCGTTGTTCTCGACGAGGCACTCCTCAAGAACCTCGACAATGGTCTTACTGGCTCCTTCCCTTGTGGTGAAAGTTGTGTCTGGATTCCATGCCTCACTGGTCCGCTCGGGTGTTCTTGCAAGAACAAAGTTTGCTACTATAACTCTCTTGACATG

MDSKIVFVALVLIATFALPSLATFEKDFITTEAVRAILKKTNSNAMPSEDVIIALTGKTLISSVVLDEALLKNLDNGLTGSFPCGESCVWIPCLTGPLGCSCKNKVCYYNSLDM
>vija17
AGCGCTCTTGCTCGCACCAAAACCATCATCTCTAACCCTGTCATCGAAGAGGCTTTGCTTAATGGTGCCAACCTGAAAGCAAGTAATGGAATCCCCTGCGCCGAGAGCTGCGTTTTCATTCCATGCACAGTCACAGCCTTACTTGGGTGCTCATGCAGCAGCAAAGTTTGCTACAACTCTCTCCAAACCAAGTAC

SALARTKTIISNPVIEEALLNGANLKASNGIPCAESCVFIPCTVTALLGCSCSSKVCYNSLQTKY
>vija18
CTCCCCGCTGCCTTTGCAGCTGAGCAAGATCTCATCACTCTCCAAGCTTATGAGGAGCTTCTCAAGAATGGGGCTGCTAATGGAATGACCAAAACTGTCATCTCAAGCCCTGTTCTTGAAGAGGCTCTCGTCTCTTACTCCAAGAACAAGCTCGGCGGTATGCCTGTCTGCGGAGAGACTTGCGTGACGGGATCATGCTACACCCCTGGGTGCTCCTGCTCCTGGCCAGTCTGCACCCAAAACTCTCTTGAAAGCACCAAATCAGCAAACCCTCTCCTTGAAGAGGCGCTCACCGCGTTCGCCAAGAAAGGTCTTGGTGGTCTTCCTGTCTGCGGTGAGACTTGCGTCGGCGGAACATGCAACACTCCTGGGTGC

LPAAFAAEQDLITLQAYEELLKNGAANGMTKTVISSPVLEEALVSYSKNKLGGMPVCGETCVTGSCYTPGCSCSWPVCTQNSLESTKSANPLLEEALTAFAKKGLGGLPVCGETCVGGTCNTPGC
>vija19
GATGTGATATCTGCTGCAGCACTGCAGGCTGTCCTTAACAGGAAAGCTCCCCTCTCCAACACCATGATGGATAACGACGCGATCGTGAATTATATCACCAGCGTCAAGACCGTCATCTCAGATCCCGCTCTCGAAGAGGCGCTGTTTAAGACCAACCATGGTGGTAATGGCTGGCCCTGCGTGGAAACTTGTATCTTTGCTAATTGGTGCGCCACCTCCGTAATTGGGTGTTCTTGCCATAGAGGTGAGTGCGAAAATAACTCTATCGACAAC

DVISAAALQAVLNRKAPLSNTMMDNDAIVNYITSVKTVISDPALEEALFKTNHGGNGWPCVETCIFANWCATSVIGCSCHRGECENNSIDN
>vija20
AGCGCTCTTGCTCGCACCAAAACCATCATCTCTAACCCTGTCATCGAAGAGGCTTTGCTTAATGGTGCCAACCTGAAAGCAGGTAATGGAATCCCCTGCGCCGAGAGCTGCGTGTGGATTCCATGCACAGTCACAGCCTTACTTGGGTGCTCATGCAGCAGCAAAGTTTGCTACAACTCTCTCCAAACCAAGTAC

SALARTKTIISNPVIEEALLNGANLKAGNGIPCAESCVWIPCTVTALLGCSCSSKVCYNSLQTKY
>vija21
AACGCCAATGTCATGCCATCTAAGGATGCCATTAATGCCCTCACCGGAAAGACCGTCATTTCGAATATTGTACTCTCGGAGGCACTCCTCAAGAACCAAGACAACGGTCTTAATGGCGTCCCTTGCGGTGAAAGTTGTGTCTTCATCCCATGCCTCACCGGCGTGATCGGGTGTTCATGCAGTAGCAATGTTTGCTACCTAAACTCTCTTAACATG

NANVMPSKDAINALTGKTVISNIVLSEALLKNQDNGLNGVPCGESCVFIPCLTGVIGCSCSSNVCYLNSLNM
>vija22
GAGGCGCTCACCGCGTTCGCCAAGAAAGGTCTTGGTGGTCTTCCTACCTGCGGTGAGACTTGCTTCACCGGAGTATGCTACACTCCTGGGTGCCAATGCGACTGGCCAATTTGCACCAGAAATGCTCTTGAGATCCAGAAACCCAACCATTTGCTCGAAGAGGCACTCGTTGCATTCGCCAAGAAAGGTAACCTCGGAGGTCTTCCCGTATGCGGCGAAACTTGC

EALTAFAKKGLGGLPTCGETCFTGVCYTPGCQCDWPICTRNALEIQKPNHLLEEALVAFAKKGNLGGLPVCGETC
>vija23
ATGAAGTTGGTGGTTGGACTTGTGGTGGTGCTTGTGGCCGTTTTTGCTCTCCCGTCTGCCTTTGCATCTTCTGAAATAAAAGATGTCATCTCTCGTGAAGCATACGAGAAGCTAGTCAACAGCGGGGCCATGGAGGGCGTCGCCATGACCAAAGCCATCATTTCAAACCCAGTTCTTGAAGAAGCACTCGTTACCGATTCTAAGAACAAACCTGGTGGTAGCATCTTCAACTGTGGTGAAAGTTGCATCTTTGGTACATGCTACACTCCTGAGTGTTCTTGCGTCTACGGTGCGTGCTCCAAGAATTCTCTACAAGGCAACGACAATAAGGCAGTGAAAGATGTCATCACTCGTGACGCATACGAGAATCTTGTTAAGAGCGGTGCCATTGAGGGCATTGCCATGACCAAAACCATCGTCTCAAACCCGGTTCTTGAAGCACTCGTTACTTATTCTAAGAACAAACTTGGTGGTAGCATCTTCAACTGCGGTGAAACTTGCATCTTGGGTACATGCTACACTCCTGGGTGTTCTTGCGTATATGGTGCGTGCTCCAAGAATTCTCTCGCCGCTAAT

MKLVVGLVVVLVAVFALPSAFASSEIKDVISREAYEKLVNSGAMEGVAMTKAIISNPVLEEALVTDSKNKPGGSIFNCGESCIFGTCYTPECSCVYGACSKNSLQGNDNKAVKDVITRDAYENLVKSGAIEGIAMTKTIVSNPVLEALVTYSKNKLGGSIFNCGETCILGTCYTPGCSCVYGACSKNSLAAN
>vija24
ATGGAAAGCACACACAATTTCATACTTGAAAGAAAAAAAGAAAATTTAGTCAAGCAATTATATATGGAGATGATGAAGAGAATGATTGTTGGGTTCGTGCTGGTTGCAGCCTTTGCTCTTCCAGCCTTTGCTGCAGCAACGTTCGAGAAAGATGTGATCACTCTCACGGCGGTTAGGTCCATTCTTGAGAAGGTGGGCCCCAACTCAAATATGGGGTTGCTGATCGAGGAGCAGACAATTAGCGCTCTCACCGGAAAGATCCTCATCACTAACCCACTCGTCGAAGCAGCACTCCTTAGGCCCTCCAAGGATAATGCCCTTCGTGGTGAACCCTGCGGTGAAACTTGCACCGAGAATTTTTGCGCTACTAAGTTTTTTGGGTGTTTTTGCAGCAACGGAGTTTGCATCAATGAAAATACTCTTGCCAAC

MESTHNFILERKKENLVKQLYMEMMKRMIVGFVLVAAFALPAFAAATFEKDVITLTAVRSILEKVGPNSNMGLLIEEQTISALTGKILITNPLVEAALLRPSKDNALRGEPCGETCTENFCATKFFGCFCSNGVCINENTLAN
>vija25
AATATGGATTCCAAGATTGTGTTTGTCGCCCTTGTCCTCATTGCAACCTTTGCCCTTCCATCCCTTGCAACCTTTGAGAAAGATTTCATCACCACTGAAGCTGTTAGGGCTATCCTCAAGAAAACCAACTCCAACGCCATGCCCTCAGAGGATGCCATTAATGCCCTCACCGGCAAGACCCTCATCTCGAGCGTTGTTCTCGACGAGGCACTCCTCAAGAACCTCCATAACGGTCTTAATGGCGTCGTCCCTTGTGGTGAAAGTTGTGTGTTTATTCCATGCCTCACTACTGTGATCGGGTGTTCTTGCAAGAGCAATGTTTGCTACAAAAACTCTCTTGACATG

NMDSKIVFVALVLIATFALPSLATFEKDFITTEAVRAILKKTNSNAMPSEDAINALTGKTLISSVVLDEALLKNLHNGLNGVVPCGESCVFIPCLTTVIGCSCKSNVCYKNSLDM
>vija26
CTTGAAGAGGCGCTCACCGCGTTCGCCAAGAAAGGTCTTGGTGGTCTTCCTACCTGCGGTGAGACTTGCTTCACCGGAGTATGCTACACTCCTGGGTGCCAGTGCGACTGGCCAATTTGCACAAAAAATGCTCTTGAGATCAAGAAACCCAACCATTTGCTCGAAGTGGCACTCGGTGCATTCGCCAAGAAAGGTAACCTCGGAGGTCTTCCCGTATGCGGCGAAACTTGCTTTGGTGGAACCTGTAACACCCCT

LEEALTAFAKKGLGGLPTCGETCFTGVCYTPGCQCDWPICTKNALEIKKPNHLLEVALGAFAKKGNLGGLPVCGETCFGGTCNTP
>viaj27
CCTGGGTGCTCCTGCTCCTGGCCAGTCTGCACCCAAAACTCTCTTGAAAGCACCAAATCAGCAAACCCTCTCCTTGAAGAGGCACTCATTGCATTCGCCAAGAAAGGTAACCTCGGTGGTATGCCCGTATGCGGCGAAACTTGCGTTGGTGGAACATGCAACACCCCTGGGTGCTCCTGCAGCTGGCCTGTGTGCACCAGAAACTCTCTTGCC

PGCSCSWPVCTQNSLESTKSANPLLEEALIAFAKKGNLGGMPVCGETCVGGTCNTPGCSCSWPVCTRNSLA
>vija28
ATGAAGATGTTTGTTGCCCTTGTGCTCTTTGCAGCCTTCGCTCTCCCCGCTGCCTTTGCAGCTGAGCAAGATCTCATCACTCTCCAAGCTTATGAGGAGCTTCTCAAGAATGGGGCTGCTAATGGAATGACCAAAACTGTCATCTCAAGCCCTGTTCTTGAAGAGGCTCTCGTCTCTTACTCCAAGAACAAGCTCGGCGGTGTGCCTGTCTGCGGAGAGACTTGCGTGACGGGATCATGCTACACCCCTGGGTGCTCCTGCTCCTGGCCAGTCTGCACCCAAAACTCTCTTGAAAGCACCAAATCAGCAAACCCTCTCCTTGAAGAGGCACTCACCACGTTCGCCAAGAAAGGTCTT

MKMFVALVLFAAFALPAAFAAEQDLITLQAYEELLKNGAANGMTKTVISSPVLEEALVSYSKNKLGGVPVCGETCVTGSCYTPGCSCSWPVCTQNSLESTKSANPLLEEALTTFAKKGL
>vija29
GTTCGTGCTGTTCTCAAGAAAACAAACTCCAATGAAATGCCTTCAGAGGAAGTCATTAATGCCCTCACTGGCAAGGTCCTCATCTCAAACATAGTTCTCCAGGAGGTGCTCCTCAAGAAACTCGACAACGGTCTTAAAGGCAGCACGCCTTGCGGTGAAAGTTGTGTCTGGATTCCATGCATCTCATCCGTGGTTGGGTGTTCTTGCAGCAACAAAGTTTGCTACATGAACTCTCTTCCCATG

VRAVLKKTNSNEMPSEEVINALTGKVLISNIVLQEVLLKKLDNGLKGSTPCGESCVWIPCISSVVGCSCSNKVCYMNSLPM
>vija30
GCATACGAAAATCTTGTCAAGAGCGGTGCCATTGAGGGTAGCATCACCATGACCAAAACCATCGTCTCAAACCCAATCCTTGAAGAAGCACTGGTTGCCTATTCCAACCACAAACTTGGAGGTGGCACCATTTTCGACTGTGGTGAAACTTGCCTTCTCGGTACATGCTACACTCCTGGTTGTTCTTGCGGCGATTACAAATTATGCTATGGCACAAATTCTCTAGAACAAAACAGCAACGAGGCAAAGGAAGAGGCACAGCTCGAAAGGGCACTCGTTACTTTCGCTAAGAAAGGTCTTGGTGGTGTTCCCATTTGCGGAGAATCTTGCATCCAGGGTACATGCTACACACCTGGGTGCACTTGCAACTGGCCTGTTTGCGAGAGAAACGCTCTTGCTATT

AYENLVKSGAIEGSITMTKTIVSNPILEEALVAYSNHKLGGGTIFDCGETCLLGTCYTPGCSCGDYKLCYGTNSLEQNSNEAKEEAQLERALVTFAKKGLGGVPICGESCIQGTCYTPGCTCNWPVCERNALAI
>vija31
ATGGATGCCCAAAAGATGAAGATGGTGATCGGTCTTGTGCTCGTCGCCACCGCGGCTTTTGCTCTGATGATCCCTGCAGCATCTGCAGTGGACGATTTCATCACTCGTCGAGCATACGACAATCTTGTCAAGAGCGGTGCCATCAATGACATTCCAATCATGGCCAAAACCATCATCTCAAACCCAGTCCTTGAAGAAGCACTGGTTACTTATTACTCTAACAACAAACTCGGCGGTAGCGTCACTGGATGTGGGGAAACTTGCTTCAAATTCAAATGCTTCACTCCTGGGTGTAAATGCGCTAAATATCCCCTCTGCTCAAAAAATTCTCTAGACGACAGTACCACGGCTGATGAGGAGGCAGTATTCGTTGCTGCGTTTCCCAGGAAACCTCTTGCAGGTCTTAATGCTAAC

MDAQKMKMVIGLVLVATAAFALMIPAASAVDDFITRRAYDNLVKSGAINDIPIMAKTIISNPVLEEALVTYYSNNKLGGSVTGCGETCFKFKCFTPGCKCAKYPLCSKNSLDDSTTADEEAVFVAAFPRKPLAGLNAN
>vija32
GCATACGAAAATCTTGTCAAGAACGGTGCCATTGAGGGCAGCATCACCATGACCAAAACCATCGTCTCAAACCCAATCCTTGAAGAAGCACTGGTTGCCTATTCCAACCACAAACTTGGAGGTGGCACCATTTTCGACTGTGGTGAAACTTGCCTTCTCGGTACATGCTACACTCCTGGTTGTTCTTGCGGCGATTACAAATTATGCTATGGCACAAATTCTCTAGAACAAAACAGCAACGAGGCAAAGGAAGAGGCACAGCTCGAAAGGGCACTCGTTACTTTCGCTAAGAAAGGTCTTGGTGGTGCTCCCATTTGCGGAGAAACTTGCTTCCAGGGTGCATGCTACACCCCTGGGTGCACTTGCGACTGGCCTGTTTGCAAGAGAAACGCTCTTGCTATT

AYENLVKNGAIEGSITMTKTIVSNPILEEALVAYSNHKLGGGTIFDCGETCLLGTCYTPGCSCGDYKLCYGTNSLEQNSNEAKEEAQLERALVTFAKKGLGGAPICGETCFQGACYTPGCTCDWPVCKRNALAI
>vija33
GCATACGAAAATCTTGTCAAGAACGGTGCCATTGAGGGCAGCATCACCATGACCAAAACCATCGTCTCAAACCCAATCCTTGAAGAAGCACTGGTTGCCTATTCCAACCACAAACTTGGAGGTGGCACCATTTTCGACTGTGGTGAAACTTGCCTTCTCGGTACATGCTACACTCCTGGTTGTTCTTGCGGCGATTACAAATTATGCTATGGCACAAATTCTCTAGAACAAAACAGCAACGAGGCAAAGGAAGAGGCACAGCTCGAAAGGGCACTCGTTACTTTCGCTAAGAAAGGTCTTGGTGGTGCTCCCATTTGCGGAGAAACTTGCTTCCAGGGTGCATGCTACACCCCTGGGTGCACTTGCGACTGGCCTGTTTGCAAGAGAAACGCTCTTGCTATT

AYENLVKNGAIEGSITMTKTIVSNPILEEALVAYSNHKLGGGTIFDCGETCLLGTCYTPGCSCGDYKLCYGTNSLEQNSNEAKEEAQLERALVTFAKKGLGGAPICGETCFQGACYTPGCTCDWPVCKRNALAI
>vija34
ATGACCAAAGCCATCATTTCAAACCCAGTTCTTGAAGAAGCACTCGTTACCGATTCTAAGAACAAACCTGGTGGTAGCATCTTCAACTGTGGTGAAAGTTGCATCTTTGGTACATGCTACACTCCTGAGTGTTCTTGCGTCTACGGTGCGTGCTCCAAGAATTCTCTACAAGGCAACAATAATAAGGCAGTGAAAGATGTCATCACTCGTGACGCATACGAGAATCTTGTTAAGAGCGGTGCCATTGAGGGCATTGCTATGACCAAAACCATCGTCTCAAACCCAGTTCTTGAAGCACTCGTTAATTATTCTAAGAACAAACTTGGTGGTAGCATCTTCAACTGCGGTGAAACTTGCATCTTGGGTACATGCTACACG

MTKAIISNPVLEEALVTDSKNKPGGSIFNCGESCIFGTCYTPECSCVYGACSKNSLQGNNNKAVKDVITRDAYENLVKSGAIEGIAMTKTIVSNPVLEALVNYSKNKLGGSIFNCGETCILGTCYT
>vija35
CTTGTAGGGCTTGTGCTCATTGCAACCTTTCCACTTCCATCTCTTGCAACCTTTGAGAGAGATATCATCACACCTGAAGCTATTCAAGCTTTTCTTGATCAGAAGAAAACCAACCCCAACTCCAATTACATGCTCTCAGACTCAGAGGATGCCGTTAATGCCCTCACCGGCAAGATCGTCATCGCTGACAACCTCTTTCTTGAGGAGGCGCTCAGGATGATGAGGAGTAATAATGTCAAGTTGGATGGAATCCACTGCGCGGAAACTTGTTTATGGGGTACATGCCGCACTGCAATCATTGGGTGTTCTTGCGAGAACAGAATTTGTTACAAGAACTCTCTTCTTGCCAAC

LVGLVLIATFPLPSLATFERDIITPEAIQAFLDQKKTNPNSNYMLSDSEDAVNALTGKIVIADNLFLEEALRMMRSNNVKLDGIHCAETCLWGTCRTAIIGCSCENRICYKNSLLAN
>vija36
GGTGCTATTGAGGGCATCACCATGACCAAAACCATCATCTCAAACCCAATCCTTGAAGAAGCACTGGTTGCCCATTTCAACCAAAAACTTGGAGGTGGTACCGGTACCATTTTCGACTGTGGTGAAACTTGCGCTTGGGGTAAATGCTACACTCCTCATTGTAGTTGCGGCAAGTATTTTTTCTGCTATGGCACAGATTCTCTAGAAAAAAACAGCAACAAGGCAGGGGAAGAGGCACTCGTTGCTTTTGCCAGGAAAGGTCTTGCAGCTGCTCTTGCTAAT

GAIEGITMTKTIISNPILEEALVAHFNQKLGGGTGTIFDCGETCAWGKCYTPHCSCGKYFFCYGTDSLEKNSNKAGEEALVAFARKGLAAALAN
>viba23
AACACAAAACACAGCTCCATCAGCAAAAGCAAAAATCAATTTTCAGAATCCATGGCAGGTACCAAGAAGATGCTTGTTGGGATTTTGCTTCTGATCGCTGCTGCTCTTCCGGCGGCGATGGCATATTCCAGCTCGTTCGAGAAAGACTTCATAACTCCTGAAGCTGTTGAGTCTGTGCTGAAGAGGAAGGCTCCTCTCTCTAACATGATGATGGAAGATGAGGATGTAGTTCTCAAAATCATCTCCACCACAAAGACCATCATCTCGAACCCAGTTCTTGAAGCGGCTATGTTGAACAACTTCAACAGTGCTAATGGCATCCCATGTGGTGAGAGCTGTGTTTGGATTCCATGCTTTAGCGCTGCTATTGGGTGTTCTTGCAGTAGCAAAGTCTGCTACAGAAACTCTCTCGATCATAAGTAC

NTKHSSISKSKNQFSESMAGTKKMLVGILLLIAAALPAAMAYSSSFEKDFITPEAVESVLKRKAPLSNMMMEDEDVVLKIISTTKTIISNPVLEAAMLNNFNSANGIPCGESCVWIPCFSAAIGCSCSSKVCYRNSLDHKY
>cycloviolacinO8
ATGGAGATGAAGAATGTGGTTGTTGGGCTCTTGCTCATTGCTGCCTTTGCTCTTCCAGCCCTTGCAACAAGCTACGAGAAAGATTTCATCACACATGAAACCGTTCAGGAAATTCTTAAGAAGGTTGGTTCCAGTTCAAACGGAATGCTAAATGAGCAAACCATCAGTGCTCTCACCAGCAAAACCATCATCTCAAACCCACTTCTTGAAGAGGCACTCTTCAAGCACTCCAACAGTATCAATGCTCTCGGTGGCACCCTTCCATGCGGTGAAAGTTGTGTGTGGATTCCTTGCATTTCCTCCGTTGTTGGGTGTTCTTGCAAGAGCAAAGTTTGCTACAAGAACTCTCTTGCT

MEMKNVVVGLLLIAAFALPALATSYEKDFITHETVQEILKKVGSSSNGMLNEQTISALTSKTIISNPLLEEALFKHSNSINALGGTLPCGESCVWIPCISSVVGCSCKSKVCYKNSLA
>Viba10	
CTCTCAAACATCATGTTAGAGGAAGATGCCATGAGTGCTCTTATCAAGAGCAAGACCGTCATCTCCAACCCAGTTATCGAAGAGGCACTCCTCAAGAACAGTAACAGTCTTCACGGCATCCCTTGTGCTGAAAGTTGTGTATATCTTCCATGCGTGACTATTGTTATTGGGTGTTCTTGCAAGGACAAAGTTTGCTACAACTCTCTTGATATA

LSNIMLEEDAMSALIKSKTVISNPVIEEALLKNSNSLHGIPCAESCVYLPCVTIVIGCSCKDKVCYNSLDI
>vibe12
ATAACAAATCAGTCAATCAATATTATTATGGAGAGCAACAAGAAGATGCAGCTTGTTGGGTTTGTGCTTCTGGCCGCCTTTGCGCTACCAGCACTTGCATCATCTTTCGAGAAAGATGTGATCTCTCCCCGTGCCATCCAGGCTGTTCTGGAGAAGAAGGGATTAAGCAAGCTGGAGGATGATCCTGTCTTGAGCGCTCTGGCTCACACCAAAACCATTATTTCTAACCCTATCATCGAAGAGGCTTTGTTTAATAGTGCCAGCCTGAATGCAGGTAATGGAGTCGCCTGCGGCGAGAGCTGCATAATCCGGCCATGCATTTTCACAGCCATACTTGGGTGCCGATGCAGGTTCACTGTTTGCGTCAACGCTCTCGAAACCAATTAC

ITNQSINIIMESNKKMQLVGFVLLAAFALPALASSFEKDVISPRAIQAVLEKKGLSKLEDDPVLSALAHTKTIISNPIIEEALFNSASLNAGNGVACGESCIIRPCIFTAILGCRCRFTVCVNALETNY
>vibe4
ATGGATGCCAAAAAGATGTTGGTTGCTCTTGTGCTTATTGCCATCTTTGCAGCCATTCCATCTCTTGCAACCTTTGAAAAGGATGTCATAACCCCTGAAGCCATTCAGGCTGTTCTTAAGAAAACCAATTACCCCAACTCTAATGTTAAGCTCTCAGAGGATGCCATTAATGCCCTGAGTAGCAAGACTGTCATCTCAAACAAAGTTCTTGAAGAGGCACTCTTCAAGATTAACACTATCAACAATGGCATTAGTGACGTCGTTAATGGCTTCCCCTGCGGGGAAAGTTGCGTCTATATTCCATGCCTCACTGCCGCTATTGGGTGTTCTTGCAAGAACAAAGTTTGCTACAAGAACTCTCTTTCCAAC

MDAKKMLVALVLIAIFAAIPSLATFEKDVITPEAIQAVLKKTNYPNSNVKLSEDAINALSSKTVISNKVLEEALFKINTINNGISDVVNGFPCGESCVYIPCLTAAIGCSCKNKVCYKNSLSN
>Viba24
ATGGAGATGAAGAAGATGATTGTTGGGTTTTTGGTGATCGCTGCCTTTGCTCTTCCAGCCTTTGCAACCGTTGAGAAAGATTTCATCACTCCTGAAGCCATTAAGATGATTTCCGGGAAGGCAAATCTCAATTTGGACGAGAAGGTCATTAGTGCTCTCACCGGTAAGATCCTCATCTCCAACCCAGTCATCGAAGAAGCACTCCTTCAGCACTCCAACTTTAATGCCCTCGGGGGCAAGATCCCTTGCGGAGAAAGTTGCGTGTGGATTCCTTGCATTACTACCGTGGTTGGTTGCTCTTGCTCGAACAAAGTTTGCTACAAGAACTCTCTTGCCAAC

MEMKKMIVGFLVIAAFALPAFATVEKDFITPEAIKMISGKANLNLDEKVISALTGKILISNPVIEEALLQHSNFNALGGKIPCGESCVWIPCITTVVGCSCSNKVCYKNSLAN
>vibe24
GCATACGAAAATCTTGTCAAGAGCGGTGCCATTGAGGGTAGCATCACCATGACCAAAACCATCGTCTCAAACCCAATCCTTGAAGAAGCACTGGTTGCCTATTCCAACCACAAACTTGGAGGTGGCACCATTTTCGACTGTGGTGAAACTTGCTTTCTTGGTAAATGCTACACTCCTGGTTGTTCTTGTGGCGAGTACAAAGTTTGCTATGGCACAAATTCTCTAGAACAAAACAGCAACGAGGCAAAGGAAGAGGCACAGCTCGAAAGGGCACTCGTTACTTTCGCTAAGAAAGGTCTTGGTGGTGTTCCCATTTGCGGAGAATCTTGC

AYENLVKSGAIEGSITMTKTIVSNPILEEALVAYSNHKLGGGTIFDCGETCFLGKCYTPGCSCGEYKVCYGTNSLEQNSNEAKEEAQLERALVTFAKKGLGGVPICGESC
>vibe19
ATGAAGATGGTGGCCGGTAACGTGCTTGTGGTGGCCGCCGCGGCCTTTGCTCTGATCCCTGCATCCTTTACAGTGGAAGATGTCATCACTCACGATCCGAACGGGAATCTTGTTACAAGCAGAGCCATTGATGGCATTATCATGACCAAAACCGTCATCTCAAACCCAATCTTTGAAGAAGCACTGCTTACCTATTCCATCAACAAACTTGGTGATCGTGCCGTTTGTGGGGAAACTTGCTTTACGGGTATATGCTACACTCCTATTTGTGTTTGCGGCAAATGGGACTTATGCCGCATGAATTCTATACAGCAAGTT

MKMVAGNVLVVAAAAFALIPASFTVEDVITHDPNGNLVTSRAIDGIIMTKTVISNPIFEEALLTYSINKLGDRAVCGETCFTGICYTPICVCGKWDLCRMNSIQQV
>Viba25	
ATGGAGATGAAGAGGATGATTGTTGGGCTTGTGCTCATCGCCACTTTTGCTCTTCCAGCCCTTAATGCAACTTTTGAGAAAGATTTCATCACTCGTCAAGCCATTAACATGGTTTTGAAGAAGGCGAGTCCCAACTCGAATGCGATGCTCGATGAGCAAGCCATTATCACTCTTACAGGCAAGATCCTCGTCTCCAACCCAATCATTGAAGAAGCTCTCCTTAAACACTCCAATCTTAATGGTCTTGGCCGCGTTCCTTGTGGCGAAAGTTGCGTCTATATCCCTTGCTTTACTAGCATTGCTGGGTGTTCTTGCAGCGACAAGGTTTGCTGGCACAACTCTCTTGCAGCCAAC

MEMKRMIVGLVLIATFALPALNATFEKDFITRQAINMVLKKASPNSNAMLDEQAIITLTGKILVSNPIIEEALLKHSNLNGLGRVPCGESCVYIPCFTSIAGCSCSDKVCWHNSLAAN
>vibe18
GTGGCCGCTTTTGCTCTCCCTACAGCCTTTGCATCTCTTGACATTAAAGATGTCATCACTCGTGAAGCATACGAGAATCTCGTTAAGAGCGGGGCCATTCAGGGCAATGCCATGACCAAAACCATCATCTCAAACCCGATCCTTGAAGAAGCACTGTCTACCTATGCCAAAAAAAAACTTGGTGGTAGCATTTTCAACTGCGGTGAAACTTGCGTTTTTGGTACGTGCTATACTCCCGGGTGTTCTTGCGTCTATGGCGCGTGCTCCAAGGATTCTCTACCAGACAGCAACAACAACAAGGCAGTGGTGAGCTCCAACTCTAGATCTAACCCGATCCTTGAAGAAGCACTCGTTTCTCTCGCTATA

VAAFALPTAFASLDIKDVITREAYENLVKSGAIQGNAMTKTIISNPILEEALSTYAKKKLGGSIFNCGETCVFGTCYTPGCSCVYGACSKDSLPDSNNNKAVVSSNSRSNPILEEALVSLAI
>vibe7
ATGGATGCCAAGAAGATGTTTCTTGCTCTTGTTCTCATTGCAACCTTTGCAGTGATTCCATCTCTTGCAACCTTTGAGAAAGATTTCATCACCCGAGAAGCCGTTCAGGCTATCCTTAAGAAAAGTGCCCCACTCTCAAACATCATGTTAGAGGAAGATATCATGAGTGCTCTTATCAAGAGCAAGACCGTCATCTCCAACCCAGTTATCGAAGAGGCACTCAGCAGGAACAGTAACGGTCTTAAGGCCGCCGTGCCTTGCGGTGAGAGTTGTGTGTGGATTCCATGCGTGACTTCTGTTGTGGGGTGTTCTTGCAGTAACAAAGTTTGCTACAACTCTCTTATA

MDAKKMFLALVLIATFAVIPSLATFEKDFITREAVQAILKKSAPLSNIMLEEDIMSALIKSKTVISNPVIEEALSRNSNGLKAAVPCGESCVWIPCVTSVVGCSCSNKVCYNSLI
>Viba11
ATGTTTGTTGCCCTTGTTCTCGTTGCAACCTTTGTCCTTCCATCTCTTGCAACCTTTGAGAAAGATTTCATCACCCCTGAAACCATTCAGGCTATCCTTAAGAAAAGTGCCCCACTCTCAAATATCATGTTAGAAGAAGATGTGATTAATGCTCTCCTCAAGAGCAAGACCGTCATCTCTAACCCAATCATCGAAGAGGCATTCCTGAAGAACAGTAATGGTCTTAATGGTATCCCTTGCGGTGAAAGTTGTGTTTGGATTCCATGCATCTCTGGTGCCATTGGGTGTTCCTGCAAGAGCAAAGTTTGCTACAGGAACTCTCTTGATAAC

MFVALVLVATFVLPSLATFEKDFITPETIQAILKKSAPLSNIMLEEDVINALLKSKTVISNPIIEEAFLKNSNGLNGIPCGESCVWIPCISGAIGCSCKSKVCYRNSLDN
>cycloviolacinO9
ATGGATGCCAAGAAGATGTTTCTTGCTCTTGTTCTCATTGCAACCTTTGCAGTGATTCCATCTCTTGCAACCTTTGAGAAAGATTTCATCACCCGAGAAGCCGTTCAGGCTATCCTTAAGAAAAGTGCCCCACTCTCAAACATCATGTTAGAGGAAGATGTTATGAATGCTCTTATCAAGAGCAAGACCGTCATCTCCAACCCAGTTATCGAAGAGGCACTCCTCAGGAATAGTAACGGTCTTAACGGCATCCCTTGTGGAGAAAGTTGTGTATGGATTCCATGCCTCACTTCTGCTGTTGGGTGTTCTTGCAAGAGCAAAGTTTGCTACAGGAACTCTCTTGATATA

MDAKKMFLALVLIATFAVIPSLATFEKDFITREAVQAILKKSAPLSNIMLEEDVMNALIKSKTVISNPVIEEALLRNSNGLNGIPCGESCVWIPCLTSAVGCSCKSKVCYRNSLDI
>cycloviolacinO12	
ATGAAGATGTTTGTTGCCCTTGTGCTCTTTGCAGCCTTCGCTCTCCCCGCTGCCTTTGCAGCTGAGCAAGATCTCATCACTCTCCAAGCTTATGAGGAGCTTCTCAAGAATGGGGCTGCTAATGGAATGACCAAAACTGTCATCTCAAGCCCTGTTCTTGAAGAGGCTCTCGTCTCTTACTCCAAGAACAAGCTCGGCGGTTTGCCTGTCTGCGGAGAGACTTGCACGTTGGGAACATGCTACACCGCTGGGTGCTCCTGCAGCTGGCCAGTCTGCACCAGAAACTCTCTTGAAAGCACCAAATCTGCAAACCCTCTCCTTGAAGAGGCACTCACCACGTTCGCCAAGAAAGGTCTTGGTGGTCTTCCCGTCTGCGGTGAGACTTGCGTCGGCGGAACATGCAACACCCCTGGGTGCACTTGCAGCTGGCCAGTCTGCACGAGAAACTCTCTTGAAAACACCAAATCAGCAAACCCTCTCCTTGAGGAGGCACTCATTGCATTCGCCAAGAAGGGTAACCTCGGCGGTCTGCCTATCTGCGGCGAAACTTGCGTTGGTGGAACATGCAACACCCCTGGGTGCTCCTGCAGCTGGCCTGTGTGCACCAGAAACTCTCTTGCCATG

MKMFVALVLFAAFALPAAFAAEQDLITLQAYEELLKNGAANGMTKTVISSPVLEEALVSYSKNKLGGLPVCGETCTLGTCYTAGCSCSWPVCTRNSLESTKSANPLLEEALTTFAKKGLGGLPVCGETCVGGTCNTPGCTCSWPVCTRNSLENTKSANPLLEEALIAFAKKGNLGGLPICGETCVGGTCNTPGCSCSWPVCTRNSLAM
>vibe17
ATGATGAAGATGTTTGTTGCCATTGTGCTCTTTGCAGCTTTTGCTCTTCCGGCTGCTTCTGCAAGTGCTTATCAAGACGTCATCACTCTCAAAGCTTACGAGGAGCTTCTCAAGAGTGGGGCTTCTACCGATGGAATGACCAAAACCGTCATCTCGAACCCTCTTCTTGAAGAGGCTCTCGTCTCCTACTCCAAGAACAAACTTGGTGGTATTGTCTATTGCGGAGAAACTTGCGGTGGTACAAGATGCTACACCCCAGGGTGTTCTTGCCGTTATCCTTACTGCAGCAAAAATTCTCTTGCCATG

MMKMFVAIVLFAAFALPAASASAYQDVITLKAYEELLKSGASTDGMTKTVISNPLLEEALVSYSKNKLGGIVYCGETCGGTRCYTPGCSCRYPYCSKNSLAM
>cycloviolacinY5	
TTAATAAGAGCTACAGCTAAAAGCATAACAAATCAGTCAATCAATATTATTATGGAGAGCAACAAGAAGATGCAGCTTGTTGGGTTTGTGCTCCTGGCCGCCTTTGCGCTACCAGCACTTGCATCATCTTTTGAGAAAGATGTGATCTCTCCCCATGCCATCCAGGCTGTTCTTGAGAAGAGGGGATTCAGCAAGCTGGAGGATGATCCTGTCGTGAGCGCTCTTGCTCGCACCAAAACCATAATCTCTAACCCTGTCATCGAAGAGGCTTTGCTTAATGGTGCCAACCTGAAAGCAGGTAATGGAATCCCCTGCGCCGAAAGCTGCGTGTGGATTCCATGCACAGTCACAGCCTTAGTTGGGTGCTCATGCAGCGACAAAGTTTGCTACAACTCTCTCCAAACCAAGTAC

LIRATAKSITNQSINIIMESNKKMQLVGFVLLAAFALPALASSFEKDVISPHAIQAVLEKRGFSKLEDDPVVSALARTKTIISNPVIEEALLNGANLKAGNGIPCAESCVWIPCTVTALVGCSCSDKVCYNSLQTKY
>cycloviolacinO22	
GGCTGCAGCTGGCCAGTCTGCACCAGAAACTCTCTTGAAAGCACCAAATCTGCAAACCCTCTCCTTGAAGAGGCACTCACCGCGTTCGCCAAGAAAGGTCTTGGTGGTCTTCCCATATGCGGTGAGACTTGCGTCGGCGGAACATGCAACACCCCTGGGTGCACTTGCAGCTGGCCAGTCTGCACGAGAAACTCTCTTGAAAACACCAAATCAGCAAACCCTCTC

GCSWPVCTRNSLESTKSANPLLEEALTAFAKKGLGGLPICGETCVGGTCNTPGCTCSWPVCTRNSLENTKSANPL
>kalataB1	
ATGAAGATGTTTGTTGCCCTTGTGCTCTTTGCAGCCTTCGCTCTCCCCGCTGCCTTTGCAGCTGAGCAAGATCTCATCACTCTCCAAGCTTATGAGGAGCTTCTCAAGAATGGGGCTGCTAATGGAATGACCAAAACTGTCATCTCAAGCCCTGTTCTTGAAGAGGCTCTCGTCTCTTACTCCAAGAACAAGCTCGGCGGTTTGCCTGTCTGCGGAGAGACTTGCACGTTGGGAACATGCTACACCGCTGGGTGCTCCTGCAGCTGGCCAGTCTGCACCAGAAACTCTCTTGAAAGCACCAAATCTGCAAACCCTCTCCTTGAAGAGGCACTCACCACGTTCGCCAAGAAAGGTCTTGGTGGTCTTCCCGTCTGCGGTGAGACTTGCGTCGGCGGAACATGCAACACCCCTGGGTGCACTTGCAGCTGGCCAGTCTGCACGAGAAACTCTCTTGAAAACACCAAATCAGCAAACCCTCTCCTTGAGGAGGCACTCATTGCATTCGCCAAGAAGGGTAACCTCGGCGGTCTGCCTATCTGCGGCGAAACTTGCGTTGGTGGAACATGCAACACCCCTGGGTGCTCCTGCAGCTGGCCTGTGTGCACCAGAAACTCTCTTGCCATG

MKMFVALVLFAAFALPAAFAAEQDLITLQAYEELLKNGAANGMTKTVISSPVLEEALVSYSKNKLGGLPVCGETCTLGTCYTAGCSCSWPVCTRNSLESTKSANPLLEEALTTFAKKGLGGLPVCGETCVGGTCNTPGCTCSWPVCTRNSLENTKSANPLLEEALIAFAKKGNLGGLPICGETCVGGTCNTPGCSCSWPVCTRNSLAM
>kalataS
CAGAAACCCAACCATTTGCTCGAAGAGGCACTCGTTGCATTCGCCAAGAAAGGTAACCTCGGAGGTCTTCCCGTATGCGGCGAAACTTGCGTTGGTGGAACCTGTAACACCCCTGGGTGCTCTTGCAGCTGGCCTGTTTGCACCAGAAATGCTCTTGAGATCCAGAAACCCAACCATTTGCTCGAAGAGGCACTCGTTGCATTCGCCAAGAAAGGTAACCTCGGAGGTCTTCCCGTATGCGGCGAAACTTGC

QKPNHLLEEALVAFAKKGNLGGLPVCGETCVGGTCNTPGCSCSWPVCTRNALEIQKPNHLLEEALVAFAKKGNLGGLPVCGETC
>Mra30
ATGTGGAGACCGATGGGCTATAAATTGAGGCTAAACTTCCACATAAATCTCACACAGCGTTTGCAATCACAGTTAAAGAAAAGGCATATGCACATCATTATGGCAAGCCAAAAGATTGTTGTTGCTTTTTTGCTCATTGCAGCTTTTGCTCTTCCTGCCTTTGCATCTTTCCAGAAAGATGTGATCACTCCCGCAGCAATGGAGGCTGTCCTTAACAGGAAAGCTCCCCTCTCCAACATCATGATGGAGAACGACGCGATCATGAATGTTATCGCCAACGTCAAGACCGTCATCTCAAATCCCGTGCTCGAAGAGGCGTTGCTTAAGACCAACCATGGTGTTAATGGCATCCCCTGCGGTGAAAGTTGTGTCTTTATTCCGTGCCTCACCTCCGCAATTGGGTGTTCTTGCAAAAGCAAAGTTTGCTACAGGAACTCTCTCGACAAT

MWRPMGYKLRLNFHINLTQRLQSQLKKRHMHIIMASQKIVVAFLLIAAFALPAFASFQKDVITPAAMEAVLNRKAPLSNIMMENDAIMNVIANVKTVISNPVLEEALLKTNHGVNGIPCGESCVFIPCLTSAIGCSCKSKVCYRNSLDN
>cycloviolacinO2
TTTGAGAAAGATTTCATCACCCCAGAAGCCATTCAGGCTATCCTTAAGAAACGTGCCCCACTCTCAAACATCATGTTAGAGGAAGATATCATGAGTGCTCTTATCAAGAGCAAGACCGTCATCTCCAACCCAGTTATCGAAGAGGCACTCCTCAGGAATAGTAACGGTCTTAACGGCATCCCTTGCGGTGAAAGTTGTGTCTGGATTCCATGCATCTCTTCTGCTATTGGGTGTTCTTGCAAGAGCAAAGTTTGCTACAGGAACTCTCTTGATATA

FEKDFITPEAIQAILKKRAPLSNIMLEEDIMSALIKSKTVISNPVIEEALLRNSNGLNGIPCGESCVWIPCISSAIGCSCKSKVCYRNSLDI
>Viba43	
ATGGAGATGAAGAATATGGTTGTTGGGCTCTTGCTCATTGCTGCCTTTGCTCTTCCAGCCCTTGCAACAAGCTTTGAGAAAGATTTCATCACACCTCAAACCGTGCAGGAAATTCTCAAGAAGGTCGGTTCCAATTCAAATGGAATGCTAGATGAACAAACCATCAGTGCTCTCACCAGCAAGACGATCATCTCAAACCCACTTCTTGAAGAGGCACTCTTCAAGCACTCCAACAGTATCAATGCTCTCGGTGGCAGCTTACCTTGCGGTGAAAGTTGTGTATTCATTCCTTGCATTTCCTCCGTTATTGGGTGTGCTTGCAAGAGCAAAGTTTGCTACAAGAACTCTCTTGCT

MEMKNMVVGLLLIAAFALPALATSFEKDFITPQTVQEILKKVGSNSNGMLDEQTISALTSKTIISNPLLEEALFKHSNSINALGGSLPCGESCVFIPCISSVIGCACKSKVCYKNSLA
>vibe3	
ATGGAGATGAAGAATGTGGTTGTTGGGCTCTTGCTCATTGCTGCCTTTGCTCTTCCAGCCCTTGCAACAAGCTTTGAGAAAGATTTCATCACACCTGAAACCGTTCAGGAAATTCTTAAGAAGGTTGGTTCCAGTTCAAACGGAATGCTAAATGAGCAAACCATCAGTGCTCTCACCAGCAAAACCATCATCTCAAACCCACTTCTTGAAGAGGCACTCTTCAAGCACTCCAACAGTATCAATGCTCTCGGTGGCACCTTTCCTTGCGGTGAAAGTTGTGTATTCATTCCTTGCATTTCCTCCGTTGTTGGGTGTTCTTGCAAGAGCAAAGTTTGCTACAAGAACTCTCTTGCT

MEMKNVVVGLLLIAAFALPALATSFEKDFITPETVQEILKKVGSSSNGMLNEQTISALTSKTIISNPLLEEALFKHSNSINALGGTFPCGESCVFIPCISSVVGCSCKSKVCYKNSLA
>Viba12
CATCACTTAATAAGAGCTACAGCTAAAAGCATAACAAATCAGTCAATCAATATTATTATGGAGAGCAACAAGAAGATGCAGCTTATTGGGTTTGTGCTCCTGGCCGCCTTTGCGCTACCAGCACTTGCATCATCTTTCGAGAAAGATGTGATCTCTGCCAGTGCCATCCAGGCTGTTCTTGCGAAGAGGGGATTCAGCAAGCTGGAGGATGATCCTGTCGTGAGCGCTCTTGCTCGCACCAAAACCATCATCTCTAACCCTGTCATCGAAGAGGCTTTGCTTAATGGTGCCAACCTGAAAGCAGGTAATGGAATCCCCTGCGCCGAGAGCTGCGTGTGGATTCCATGCACAGTCACAGCCTTACTTGGGTGCTCATGCAAGGACAAAGTTTGCTACAACTCTCTCCAAACCAAGTAC

HHLIRATAKSITNQSINIIMESNKKMQLIGFVLLAAFALPALASSFEKDVISASAIQAVLAKRGFSKLEDDPVVSALARTKTIISNPVIEEALLNGANLKAGNGIPCAESCVWIPCTVTALLGCSCKDKVCYNSLQTKY
>Viba17
TCCAAGAACAAGCTCGGCGGTCTTCCTGTCTGCGGAGAGACTTGCGTCGGTGGAACATGCAACACCCCTGGGTGCGGCTGCAGCTGGCCAGTCTGCACCAGAAACTCTCTTGAAAGCACCAAATCTGCAAACCCTCTCCTTGAAGAGGCACTCACCACGTTCGCCAAGAAAGGTCTTGGCGGTCTTCCCGTCTGCGGTGAG

SKNKLGGLPVCGETCVGGTCNTPGCGCSWPVCTRNSLESTKSANPLLEEALTTFAKKGLGGLPVCGE


Table S1. All cyclotides and their sequences found in Viola japonica as well as some observed molecular weights of potentially cyclotide compounds detected in the extracts of Viola japonica by mass spectrometry. D-value=caculated mass-Observed mass. ND Indicates not detected. The theoretical molecular weights of cyclotides obtained through transcriptome sequencing may exhibit discrepancies with the actual molecular weights observed through mass spectrometry. Some compounds with lower abundance limited further purified for sequence analysis, thus leading to less accurate results. 

A

cycloviolacin O8: 1076.7991 Da(3+)       viba 24: 1090.4731 Da(3+) 
B

viba 43: 1058.4677 Da(3+)    vibe 3: 1612.1848 Da(2+)
C

viba 12: 1577.6765 Da(2+)  kalata B1,cycloviolacin O12: 1447.0599 Da(2+)  vija 9: 1480.5754 Da(2+)
D

Cycloviolacin Y5: 1550.1345(2+)   Mra30: 1558.6476(2+)


E

Cycloviolacin O22: 1454.5692 Da(2+)  
F

kalata S: 1440.0551Da(2+)      viba 17: 1425.0483 Da(2+)
G     

vija 22: 1035.2078 Da(3+)
H  

vija 4: 1080.8035 Da(3+)   
I

vija28: 972.8594 Da(3+)
Figure S1. A-I: The molecular weights obtained from mass spectrometry analysis of crude extracts from Viola japonica are shown in the figure below. (Potential cyclotides and their observed molecular weights(net charge)). Using the Thermo Orbitrap Exploris 240 mass spectrometer for mass spectrometry analysis, with a duration of 60 minutes. The mobile phase A is 0.1% formic acid water and  mobile phase B is 80% acetonitrile. The gradient elution flow rate is 300 nl/min. The concentration of mobile phase B increases to 70% within the first 35 minutes. The detection mode is positive ion, with a precursor ion scanning range of 500~2500 m/z.


Figure S2. The chromatogram of the collected solution (1%TFA in 30%ACN) of Viola japonica after purification by SPE is shown in the figure, and the peak positions of vija 12 and vija 31 are indicated. The mobile phase B consists of a 90% acetonitrile/water solution, with a liquid phase gradient that increases from 5% B to 60% B within 40 minutes.


Figure S3. Purified cyclotides chromatography and mass spectrometry data. The extraction of cyclotides from Viola japonica involved immersion in acetonitrile-water solution, followed by solid-phase extraction (SPE) crude purification and high-performance liquid chromatography (HPLC) purification. The obtained cyclotides exhibited HPLC chromatograms and mass spectra, along with post-reduced alkylation mass spectra, indicating a molecular weight increase of 348 Da.


Name	Sequence	Trypsin digestion peptide fragment	
vija 10	GSIPCGESCVFIPCISALLGCSCSSKVCYKN	NGSIPCGESCVFIPCISALLGCSCSSK	
		VCYK	
viba 11	GIPCGESCVWIPCISGAIGCSCKSKVCYRN	NGIPCGESCVWIPCISGAIGCSCK	
		SKVCYR	
cycloviolacin O2	GIPCGESCVWIPCISSAIGCSCKSKVCYRN	NGIPCGESCVWIPCISSAIGCSCK	
		SKVCYR	
vija 12	GSYISCGETCVKLKCYTPGCTCTWPACKKN	NGSYISCGETCVK	
		CYTPGCTCTWPACKK	
		LK	

Table S2. Cyclotides fragments after being degraded by trypsin. After purification, the cyclotides underwent reduction and alkylation reactions, followed by enzymatic digestion using trypsin. The resulting fragment sequences are showed in the table.


Figure S4. viba 11 Trypsin Digestion Mass Spectrum. The viba 11 was reduced by dithiothreitol and alkylated by iodoacetamide before being digested by trypsin. The fragment peptides were analyzed by ESI-MS/MS.

Table S3. b and y ion of viba 11 from trypsin digestion mass spectrum. 


Figure S5. cycloviolacin O2 Trypsin Digestion Mass Spectrum. The cycloviolacin O2 was reduced by dithiothreitol and alkylated by iodoacetamide before being digested by trypsin. The fragment peptides were analyzed by ESI-MS/MS.

Table S4. b and y ion of cycloviolacin O2 from trypsin digestion mass spectrum. 


Figure S6. vija 12 Trypsin Digestion Mass Spectrum. The cycloviolacin O2 was reduced by dithiothreitol and alkylated by iodoacetamide before being digested by trypsin. The fragment peptides were analyzed by ESI-MS/MS.

Table S5. b and y ion of vija 12 from trypsin digestion mass spectrum. 


Table S6. b and y ion of vija 10 from trypsin digestion mass spectrum. 
